# Prone position versus usual care in hypoxemic COVID-19 patients in medical wards: a randomised controlled trial

**DOI:** 10.1186/s13054-023-04529-z

**Published:** 2023-06-17

**Authors:** Mai-Anh Nay, Raphaël Hindre, Christophe Perrin, Jérémy Clément, Laurent Plantier, Aymeric Sève, Sylvie Druelle, Marine Morrier, Jean-Baptiste Lainé, Léa Colombain, Grégory Corvaisier, Nicolas Bizien, Xavier Pouget-Abadie, Adrien Bigot, Simon Jamard, Elsa Nyamankolly, Benjamin Planquette, Guillaume Fossat, Thierry Boulain

**Affiliations:** 1 Medical Intensive Care Unit, Centre Hospitalier Régional d’Orléans, 14, Avenue de l’hôpital, 45067 Orléans Cedex 2, France; 2 Department of Respiratory Medicine, Hôpital Européen Georges-Pompidou, Paris, France; 3 Innovative Therapies in Hemostasis, INSERM UMR S 1140, Biosurgical Research Lab (Carpentier Foundation), Université de Paris, Paris, France; 4 Department of Pneumology and Pneumo-Covid Unit, Centre Hospitalier Princesse Grace, Monaco, Monaco; 5 Department of Internal Medicine and General Medicine, Centre Hospitalier Simone Veil, Blois, France; 6 Department of Pneumology and Respiratory Functional Testing, Bretonneau Hospital, CHRU de Tours, CEPR/INSERM UMR1100, University of Tours, Tours, France; 7 Department of Infectious and Tropical Diseases, Centre Hospitalier Régional d’Orléans, Orléans, France; 8 Department of Pneumology, Centre Hospitalier Régional d’Orléans, Orléans, France; 9 Department of Infectious Diseases, Centre Hospitalier Departmental de la Vendée, La Roche Sur Yon, France; 10 Department of Infectious and Tropical Diseases, Le Mans Hospital, Le Mans, France; 11 Department of Infectious and Tropical Diseases, Perpignan Hospital Centre, Perpignan, France; 12 Department of Internal Medicine, Centre Hospitalier Bretagne Atlantique, Vannes, France; 13 Department of Pneumology, Centre Hospitalier Intercommunal de Cornouaille, Quimper, France; 14 Department of Internal Medicine and Infectious Diseases, Groupement Hospitalier la Rochelle Ré Aunis, La Rochelle, France; 15 Department of Internal Medicine, Bretonneau Hospital, Tours, France; 16 Department of Infectious Diseases, Bretonneau Hospital, University of Tours, Tours, France; 17 Department of Internal Medicine and Infectious Diseases, Hospital Dax Côte D’argent, Dax, France

**Keywords:** COVID-19, Awake prone position, Non-intubated, Spontaneous breathing, Respiratory failure, Hypoxemia, Medical wards

## Abstract

**Background:**

Benefit of early awake prone positioning for COVID-19 patients hospitalised in medical wards and who need oxygen therapy remains to be demonstrated. The question was considered at the time of COVID-19 pandemic to avoid overloading the intensive care units. We aimed to determine whether prone position plus usual care could reduce the rate of non-invasive ventilation (NIV) or intubation or death as compared to usual care alone.

**Methods:**

In this multicentre randomised clinical trial, 268 patients were randomly assigned to awake prone position plus usual care (*N* = 135) or usual care alone (*N* = 132). The primary outcome was the proportion of patients who underwent NIV or intubation or died within 28 days. Main secondary outcomes included the rates of NIV, of intubation or death, within 28 days.

**Results:**

Median time spent each day in the prone position within 72 h of randomisation was 90 min (IQR 30–133). The proportion of NIV or intubation or death within 28 days was 14.1% (19/135) in the prone position group and 12.9% (17/132) in the usual care group [odds ratio adjusted for stratification (aOR) 0.43; 95% confidence interval (CI) 0.14–1.35]. The probability of intubation, or intubation or death (secondary outcomes) was lower in the prone position group than in the usual care group (aOR 0.11; 95% CI 0.01–0.89 and aOR 0.09; 95% CI 0.01–0.76, respectively) in the whole study population and in the prespecified subgroup of patients with SpO_2_ ≥ 95% on inclusion (aOR 0.11; 95% CI 0.01–0.90, and aOR 0.09; 95% CI 0.03–0.27, respectively).

**Conclusions:**

Awake prone position plus usual care in COVID-19 patients in medical wards did not decrease the composite outcome of need for NIV or intubation or death.

*Trial registration* ClinicalTrials.gov Identifier: NCT04363463. Registered 27 April 2020.

**Supplementary Information:**

The online version contains supplementary material available at 10.1186/s13054-023-04529-z.

## Introduction

COVID-19 may cause pneumonia, hypoxemia and acute respiratory distress syndrome. Prone positioning can improve oxygenation in several ways [1–5], it can reduce mortality, and it is now part of the standard of care in sedated, mechanically ventilated patients with severe acute respiratory distress syndrome [6]. In critically ill COVID-19 patients requiring nasal high-flow oxygen therapy, awake prone positioning was recently shown to reduce the incidence of intubation or death and the incidence of intubation [7, 8].

Among COVID-19 patients who are hospitalised in medical wards and require oxygen therapy, the rate of intensive care unit (ICU) admission to initiate mechanical respiratory support is high [9–12]. The role of awake prone positioning in the care of these patients remains to be evaluated. Several observational studies have shown encouraging results, mainly an improvement in oxygenation [13–18]. Small randomised controlled trials (RCTs) were uninformative [12, 19, 20]. A large non-RCT found no benefit of prone positioning in hypoxemic patients [21]. One recent RCT showing no benefits of prone positioning in non-critically ill COVID-19 patients was stopped early for futility [22].

This multicentre, pragmatic RCT of COVID-19 patients requiring oxygen therapy and hospitalised in medical wards aimed to determine whether awake prone position plus usual care compared to usual care alone could reduce the incidence of non-invasive ventilation (NIV) or intubation or death. The question was considered at the surge of the COVID-19 pandemic to avoid overloading ICUs.

## Methods

### Study design

The study was conducted in 15 medical wards at 12 hospitals in France and Monaco. For all the hospitals, the study was approved by a French ethics committee (Comité de Protection des Personnes Ouest VI, Brest, France, no. 1279 HPS2) and a Monegasque ethics committee (Comité Consultatif d’Ethique en matière de Recherche Biomédicale, Monaco, no. 2020.8894 AP/jv) and complies with the current revision of the Declaration of Helsinki, the Inter-national Conference on Harmonisation Note for Guidance on Good Clinical Practice and the applicable French and Monegasque regulatory requirements. Written informed consent was obtained from all patients before inclusion. Patients were not involved in the design or planning of this study.

### Participants

Eligible patients were hospitalised in medical wards for < 72 h, were 18–85 years old, had laboratory-confirmed COVID-19 pneumonia, were breathing spontaneously with supplemental oxygen (via standard nasal prongs, mask or high-flow nasal cannula) and were able to self-position in the prone position or with the assistance of one person. Exclusion criteria included respiratory support at home, chronic obstructive pulmonary disease (Gold stage 3 or 4), contraindication to prone positioning (recent thoracic trauma, pneumothorax, unstable spine or pelvis fractures), deep vein thrombosis or pulmonary embolism with curative anticoagulation for < 48 h, respiratory rate > 40/min or excessive use of respiratory muscles (as judged by the clinician), patient discharged from ICU after having invasive intubation or NIV for COVID-19 pneumonia, a do-not-intubate order, pregnant or breastfeeding women and patient not affiliated or excluded from French or Monegasque public health insurance or under law protection (minors, persons deprived of their liberty by court or administrative decision).

### Randomisation

Patients were randomised in a 1:1 ratio by the use of a centralised web-based management system (EOL Random, Medsharing, France) to awake prone position plus usual care or usual care alone in permuted blocks (size unknown to investigators) with stratification on ward, body mass index (< or ≥ 30 kg/m^2^) and severity of oxygenation impairment. The severity of oxygenation impairment, moderate or mild, was defined according to the results of a standardised test before randomisation: oxygen saturation (SpO_2_) < or ≥ 95% after 5 min of oxygen therapy at a flow of 5 L/min delivered via a face mask or standard nasal prongs. The cut-off value of SpO_2_ < or ≥ 95% corresponds to an SpO_2_/inspired fraction of oxygen (FiO_2_) ratio of 235, assuming that FiO_2_ is roughly equal to 0.40 under 5L min^−1^ of standard oxygen [23]. This also corresponds to an arterial partial pressure of oxygen/FiO_2_ ratio of 200 mm Hg [24], a threshold that distinguishes mild from moderate COVID-19-related respiratory failure in spontaneously breathing patients [25]. The nature of the intervention precluded blinding of patients and staff members.

### Study intervention

As soon as possible after randomisation, patients assigned to the intervention group had to lie in a prone position for a minimum of two sessions with the goal of a cumulative time of at least 150 min in the prone position during the daytime. Patients were encouraged to lie in the prone position as much as possible. Time and duration of each mobilisation were recorded in a notebook by the patient or a staff member, except at night because of the extra workload of caregivers during the pandemic. Between sessions of prone position, patients could lie supine or be in the semi-sitting position in bed or in a chair. Intolerance and complications of prone position were recorded in the notebook.

Patients assigned to usual care alone had to stay in the semi-sitting position in bed (minimum 30° inclination, not more than 60°–70° inclination) or in a chair during the daytime. The prone position was not allowed during the daytime. It was allowed at night if it was the patient’s natural position of sleeping. The lateral decubitus positioning was allowed. Position during the daytime was recorded by the patient or a staff member, except at night because of the extra workload of caregivers during the pandemic.

### Outcomes

The primary outcome was the proportion of treatment failures defined by a composite endpoint consisting of the need for NIV (at two pressure levels) or intubation or death within 28 days of enrolment.

The following secondary outcomes were compared between groups: the rate of endotracheal intubation at 28 days; the rate of NIV at 28 days; the rate of a two-point decrease in the clinical World Health Organization (WHO) ordinal scale (change in patient hospitalisation status using a seven-point ordinal severity: 1—not hospitalised, capable of resuming normal activities, 2—not hospitalised but unable to resume normal activities, 3—hospitalised, no oxygen therapy, 4—hospitalised and requiring oxygen therapy, 5—hospitalised and requiring NIV or nasal high-flow oxygen, 6—hospitalised and requiring mechanical ventilation, 7—hospitalised and requiring mechanical ventilation with other organ support (vasopressors, renal replacement therapy, extracorporeal life support), and 8—dead. An improvement is a decrease in the clinical WHO scale, and a clinical worsening is defined as an increase in the clinical WHO scale) [26] from randomisation to 28 days (defined by continuous uneventful improvement, i.e. without the need for escalating respiratory support, from the date of inclusion to a two-point decrease in the clinical WHO scale); time spent with supplemental oxygen from inclusion to day 28; hospital length of stay; mortality at 28 days; and during the hospital stay; and rate of transfer to an ICU at 28 days.

### Sample size

Given scarce published data [15, 16, 19] at the time we launched the trial, the sample size calculation was highly speculative and was revised in November 2021 [27] because of the decrease in SARS-CoV-2 infection rate in France at that time. We assumed that the treatment failure rate would be 4% in the intervention group and 14% in the usual care group, with lost to follow-up < 2%. With a two-sided alpha risk of 5% and a statistical power of 80%, a total of 268 patients were required (134 in each group).

### Statistical analysis

Patients were analysed on an intention-to-treat basis unless they withdrew consent for the use of any data. Data are summarised by number of participants (percentage) for categorical variables and mean (SD) or median (IQR) for continuous variables.

The published statistical plan, which planned to use a Mantel–Haenszel Chi-squared test to analyse the primary outcome, was modified (see Additional files 1 and 2, page 3 for details) [27]. The relative risk of NIV or intubation or death within 28 days of enrolment (primary outcome) between randomisation groups was estimated by adjusted odds ratios (aOR) and 95% confidence interval (95% CI) obtained by a multivariable mixed-effect logistic regression model, with the recruiting ward as a random effect and study intervention and stratification variables (SpO_2_ and body mass index) as fixed-effect variables. Interaction terms between the stratification variables and the intervention were retained if they showed a significant statistical link with the frequency of treatment failure.

The binary secondary outcomes were compared between groups with the same method. The time to clinical improvement (two points on the WHO scale) was compared between groups by using a competing risk analysis approach (Fine and Gray model) with escalation of respiratory support as a competing event; a two-point improvement in the clinical WHO scale was defined as a continuous uneventful improvement (i.e. without the need for escalating respiratory support [including increase in oxygen flow]), from the date of inclusion to a two-point decrease.

The differences in durations (of oxygen therapy and hospitalisation) were compared by a Mann–Whitney *U* test adjusted for the stratification variables and expressed as the median difference; 95% CIs were obtained by bootstrapping (2000 unstratified samples with replacement).

A per-protocol analysis was performed in the study population restricted to patients of the intervention group who laid prone for at least 2 h each day during the daytime and patients of the usual care group who never laid prone. Days considered were those preceding the use of non-invasive ventilation, intubation, transfer to an ICU, death, weaning of oxygen therapy or discharge from the hospital, whichever occurred first.

The above analyses were repeated in prespecified subgroups formed according to the stratification variables. Post hoc time-to-event exploratory analyses were conducted (see Additional file 2, page 9 for details).

The analyses were conducted with R v. 4.0.2. (R Foundation for Statistical Computing). A two-tailed *P* < 0.05 was considered statistically significant. We did not adjust for multiple testing. Therefore, the analyses of secondary outcomes should be considered exploratory.

## Results

### Participants

**Fig. 1:**
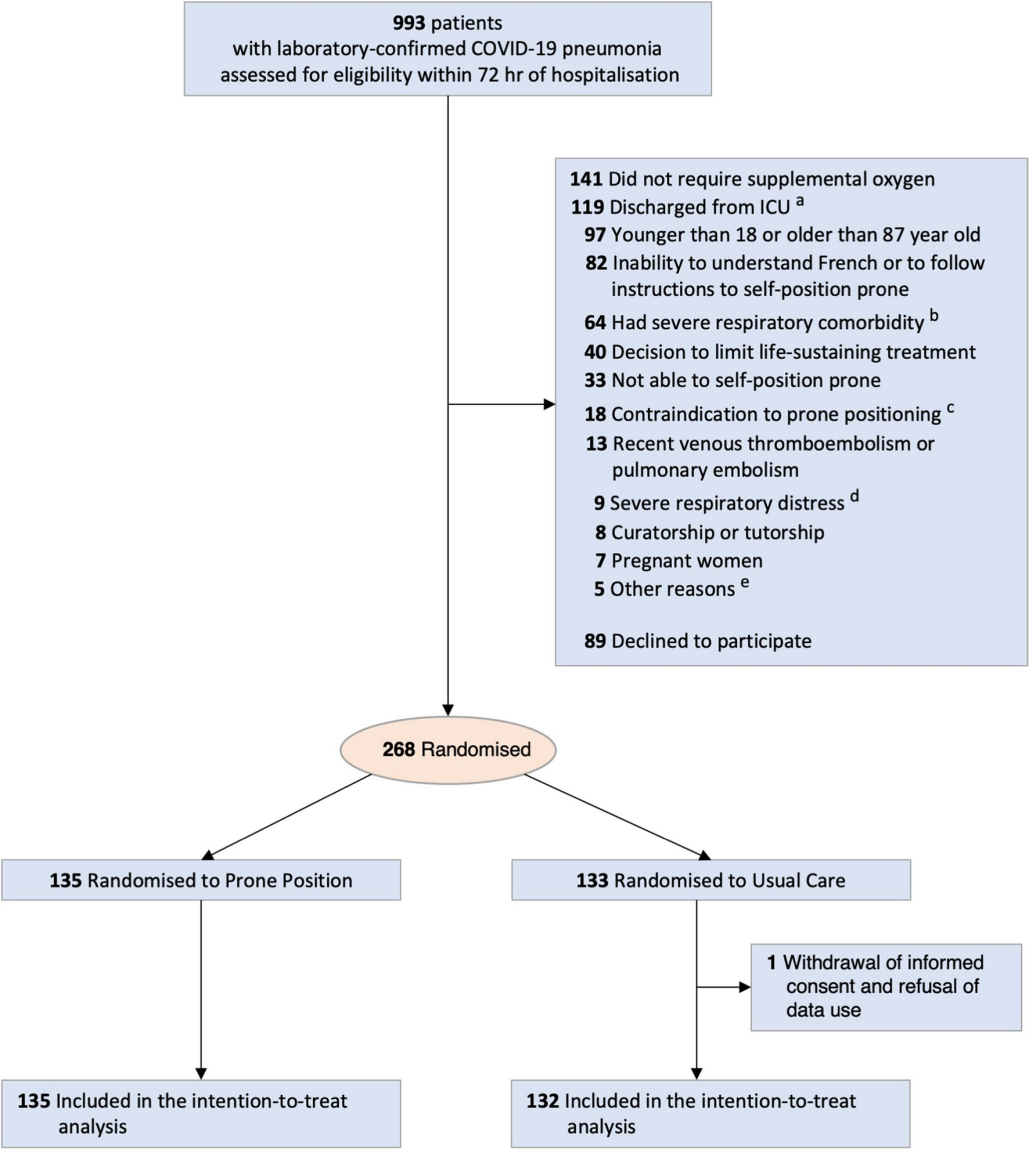
Patient recruitment flowchart. *Abbreviation* ICU, intensive care unit. ^a^Patients discharged from the ICU after non-invasive ventilation or intubation for COVID-19 pneumonia. ^b^Thirty-one patients had oxygen therapy at home; 24 had severe chronic obstructive pulmonary disease Gold stage 3 or 4; 9 had known chronic diffuse interstitial lung disease. ^c^Recent thoracic trauma, pneumothorax, unstable spine or pelvis fractures. ^d^Six patients had respiratory frequency > 40/min on screening; two showed excessive use of accessory respiratory muscles as judged by the clinician in charge; one had an indication for non-invasive ventilation for acute pulmonary oedema. ^e^Three patients had chronic neuromuscular disease; one had an intestinal occlusive syndrome on screening; one had no social insurance

Between 28 August 2020 and 5 January 2022, 993 patients were screened in 15 medical wards at 12 hospitals. Originally, 20 wards were scheduled to participate in the study, but 5 did not enrol any patient and stopped participating in the study. A total of 268 patients were randomised (Fig. 1). One patient withdrew consent, so data for 267 patients (135 in the intervention group and 132 in the usual care group) were included in the intention-to-treat analysis.

Characteristics of patients at inclusion were similar between groups (Table 1). The mean [SD] age was 59 [11.5] years, 71% of patients were male, and the mean [SD] body mass index was 28.6 [4.4]. The median SpO_2_ was 96% [IQR, 94–98] at inclusion, and the mean flow of supplemental oxygen used was 3 L/min [IQR, 2–5] for 252 patients; 12 patients were receiving nasal high-flow oxygen on inclusion (median fraction of inspired oxygen [FiO_2_] 0.84 [IQR, 00.70–1.00]. In total, 99% of patients were receiving intravenous corticosteroids therapy.

**Table 1:** Baseline characteristics of patients with COVID-19 under prone position or usual care

Characteristics	Prone position *N* = 135	Usual care *N* = 132	SMD
Age, mean (SD), y	58.4 (12.1)	59.2 (11.0)	0.065
Men	96 (71)	93 (71)	0.014
Body mass index, mean (SD), kg/m^2^	28.6 (4.2)	28.6 (4.7)	0.014
*Medical history*			
Hypertension	26 (19.3)	35 (26.5)	0.173
Coronary heart disease	9 (6.7)	5 (3.8)	0.130
Type 1 diabetes	1 (0.7)	1 (0.8)	0.002
Type 2 diabetes	11 (8.1)	18 (13.6)	0.177
Chronic obstructive pulmonary disease	6 (4.4)	1 (0.8)	0.233
Asthma	11 (8.1)	9 (6.8)	0.1051
Cerebral vascular disease	2 (1.5)	1 (0.8)	0. 069
Dialysis	0 (0)	0 (0)	< 0.0001
Liver cirrhosis	1 (0.7)	0 (0.0)	0.122
Immunosuppression^a^	8 (5.9)	4 (3.0)	0.140
*Treatment at inclusion for COVID-19*			
Corticosteroids	134 (99.3)	130 (98.5)	0.073
*Preferential sleeping position as declared by patients*			
Prone position	13 (9.6)	14 (10.6)	0.032
Other	107 (79.3)	105 (79.5)	0.007
Don’t know	8 (5.9)	4 (3.0)	0.140
Missing data	7 (5.2)	9 (6.8)	0.069
*SpO*_*2*_* at inclusion, median (IQR), %*^b^	96 (94–98)	96 (94–98)	0.019
*Oxygen therapy at inclusion*			
Standard oxygen therapy, No. (%)^c^	129 (96)	126 (95)	
Nasal high-flow oxygen therapy, No. (%)^d^	6 (4)	6 (5)	
Flow of oxygen if standard oxygen therapy, median (IQR), L/min	3 (2–5)	4 (2–6)	
PaO_2_, median (IQR), mmHg^e^	71 (64–83)	71 (62–83)	
PaCO_2_, median (IQR), mmHg^e^	34 (32–37)	34 (32–37)	
PaO_2_–FiO_2_ ratio, median (IQR), mmHg^f^	178 (151–226)	173 (131–226)	
*Stratification*^c^			
Body mass index ≥ 30 kg/m^2^ and SpO_2_ ≥ 95%	29 (21.5)	28 (21.2)	_
PaO_2_–FiO_2_ ratio, median (IQR), mmHg	200 (177–244)	188 (159–218)	
Body mass index ≥ 30 kg/m^2^ and SpO_2_ < 95%	13 (9.6)	13 (9.8)	_
PaO_2_–FiO_2_ ratio, median (IQR), mmHg	162 (114–190)	139 (99–157)	
Body mass index < 30 kg/m^2^ and SpO_2_ ≥ 95%	63 (46.7)	61 (46.2)	_
PaO_2_–FiO_2_ ratio, median (IQR), mmHg	185 (162–235)	194 (149–250)	
Body mass index < 30 kg/m^2^ and SpO_2_ < 95%	30 (22.2)	30 (22.7)	_
PaO_2_–FiO_2_ ratio, median (IQR), mmHg	152 (114–185)	133 (113–215)	

*SMD* standardised difference of means or proportions, *SpO*_*2*_ oxygen saturation measured by pulse oximetry, *PaO*_*2*_ partial pressure of oxygen in arterial blood, *PaCO*_*2*_ partial pressure of carbon dioxide in arterial blood, *FiO*_*2*_ fraction of inspired oxygen, *SD* standard deviation, *IQR* interquartile range

^a^Patients were considered immunosuppressed if they used long-term (> 3 months) corticosteroids or other immunosuppressant drugs or had solid organ transplantation, a solid tumour requiring chemotherapy in the last 5 years, haematologic malignancy or primary immune deficiency

^b^SpO_2_ was measured after inclusion during a standardised test, see Methods

^c^Supplemental oxygen was given via standard nasal prongs or face mask at a flow rate up to 15 L/min

^d^Nasal high-flow oxygen therapy was administered via large bore nasal cannula with a range of flow from 40 to 70 L/min in the prone position plus usual care group and from 30 to 60 L/min in the usual care alone group. The FiO_2_ range was from 0.4 to 1.0 in the prone position plus usual care group and from 0.8 to 1.0 in the usual care alone group

^e^Blood gases were those sampled within 24 h before inclusion; data were missing for 11 and 8 patients in the intervention and usual care groups

^f^The FiO_2_ was estimated according to Wettstein et al. [23]

All patients assigned to the prone positioning group, received at least one dose of prone positioning. 101 (74.8%) proned on the day of enrolment. 30(20%) first proned on study days 2 and 4 (3%) on study day 3. Data for time spent in the prone position each day during the daytime were missing for 19 (14.1%) patients and were not replaced. For the remaining 116 patients, the median time spent in the prone position per day during the daytime was 138 min [IQR, 90–176] during the study period, and 14 patients (12.1%) had at least 1 day without a prone position during the daytime because of intolerance or refusal. The median time spent each day in the prone position within 72 h of randomisation was 90 min [IQR, 30–133]. Two (1.5%) patients did not lie prone at all during the study period because of immediate intolerance (pain and worsening of dyspnoea). Only one occurrence of desaturation leading to immediate repositioning of the patient to the supine position was recorded. Overall, 42 patients (31.1% of the prone position group) laid prone for > 2 h each day during the daytime. In the usual care group, patients never laid in prone position during the daytime with the exception of one patient who deliberately chose to regularly lie prone from the 7th to the 12th day of hospitalisation despite regular recalls of the protocol.

### Primary outcome

In the intention-to-treat population, the rate of NIV or intubation or death within 28 days was 14.1% in the prone position group and 12.9% in the usual care group (aOR 0.43; 95% CI 0.14–1.35; *P* = 0.15) (Table 2).

**Table 2:** Primary and secondary outcomes in the prone position and usual care groups

	No. of events/total No. of patients			
	Prone position	Usual care	Adjusted OR (95%CI) ^a^	*P*-value
*Primary outcome*				
Non-invasive ventilation or tracheal intubation, or death within 28 days	19/135	17/132	0.43 (0.14–1.35)	0.15
*Secondary outcomes*				
Non-invasive ventilation within 28 days of enrolment^b,c^	12/135	8/132	4.86 (0.95–24.87)	0.057
Tracheal intubation within 28 days of enrolment	10/135	13/132	0.11 (0.01–0.89)	0.038
Tracheal intubation or death within 28 days of enrolment	10/135	14/132	0.09 (0.01–0.76)	0.027
Death within 28 days	0/135	4/132	_	_^d^
Rate of transfer to the ICU within 28 days of enrolment	21/135	20/135	1.04 (0.53–2.05)	0.91
Weaning from oxygen in hospital ward within 28 days	117/135	110/132	1.50 (0.71–3.17)	0.29
Death during hospitalisation	2/135 ^e^	4/132	0.49 (0.09–2.74)	0.41
Do-not-intubate order after inclusion within 28 days^f^	1/135	2/132	_	_^d^
Readmission in hospital after hospital discharge and within 28 days	2/135 g	0/132	_	_^d^

	Median (IQR)	Median difference (95%CI)		
Duration of oxygen therapy in patients not transferred to the ICU or undergoing non-invasive ventilation or intubation, *days*	5 (3–8)	5 (3.5–8)	0 (− 1 to 1)	.95
Length of hospitalisation, *days*	7 (5–11)	7 (7–12)	0 (− 1 to 1.5)	.79

*OR* odds ratio, *CI* confidence interval, *ICU* intensive care unit

^a^Odds-ratios were adjusted for stratification

^b^Among the 20 patients who underwent non-invasive ventilation, 7 (5 in the prone position group and 2 in the usual care group) were not transferred to an ICU. Among the remaining 13 patients, 2 (1 in each group) underwent non-invasive ventilation the day before ICU admission, and 11 within the first 2 days of ICU admission

^c^Among the 20 patients who underwent non-invasive ventilation, 25% (3/12) in the prone position group and 62.5% (5/8) in the usual care group were intubated and/or died thereafter

^d^There were too few events to allow for meaningful logistic regression analysis

^e^Two patients died during their hospitalisation after day 28

^f^All three patients died within 28 days of inclusion

^g^The two patients were readmitted to an ICU

### Secondary outcomes

The risk of intubation, or intubation or death was lower in the intervention than in the usual care group (aOR 0.11; 95% CI 0.01–0.89; *P* = 0.038 and aOR 0.09; 95% CI 0.03–0.27; *P* = 0.027, respectively). NIV in the 28 days of enrolment tended to be more frequent in the prone position than usual care group (aOR 4.86, 95% CI 0.95–24.87, *P* = 0.057) (Table 2), mostly driven by patients with a body mass index < 30 kg/m^2^ (see subgroup analyses below).

The clinical WHO scale score improved by two points during the study period in 82.2% and 82.6% of patients (*P* > 0.99) in the intervention and usual care groups, respectively. Time to improvement on the clinical WHO scale did not differ between the groups (in Additional file 2, page 6), even when adjusted for baseline characteristics that were unbalanced between groups (i.e. standardised difference > 0.1, as shown in Table 1) (in Additional file 2, page 6). The median hospital length of stay was 7 days in both groups (*P* = 0.79).

### Prespecified subgroups

**Fig. 2:**
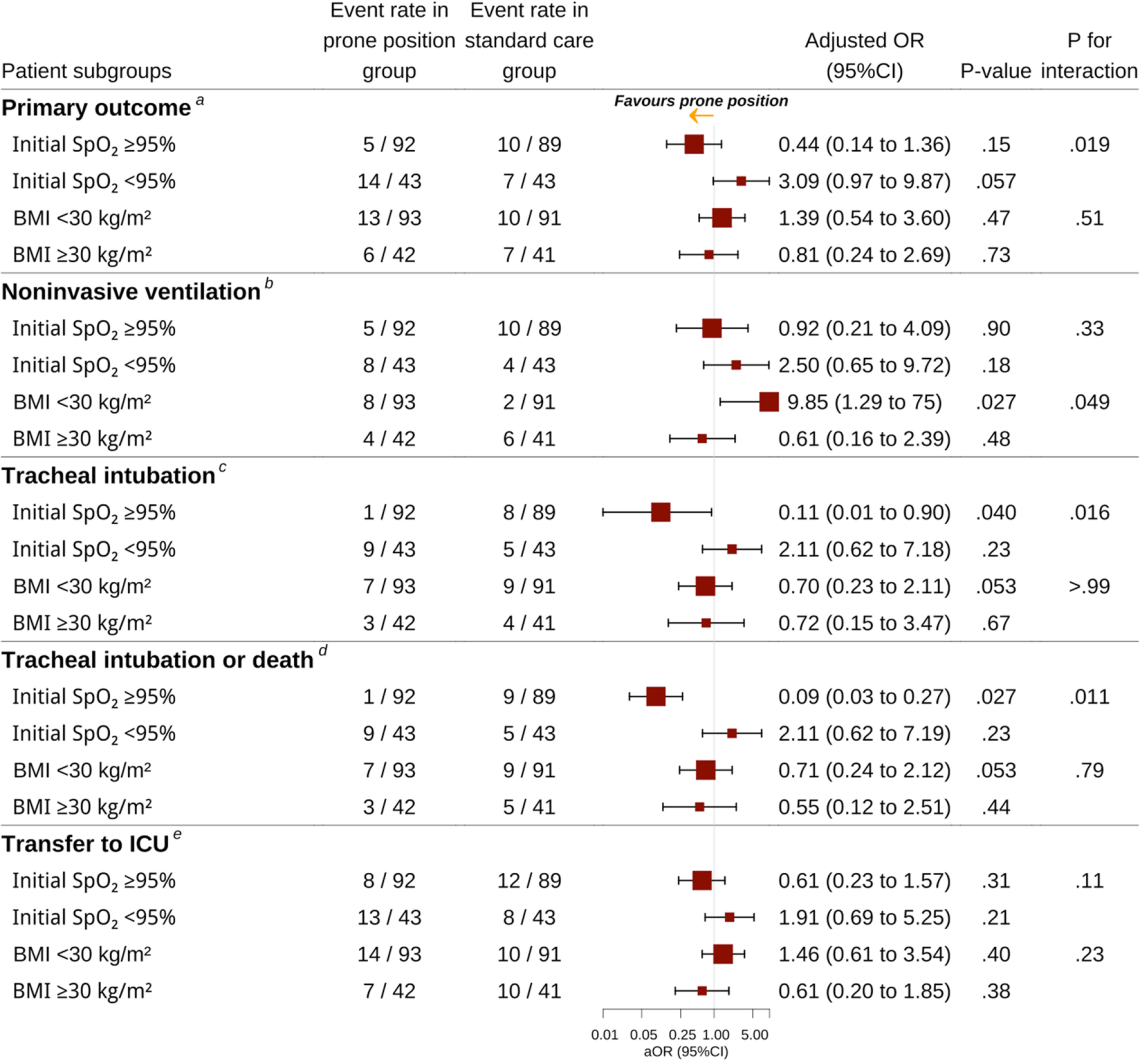
Between-group comparison of outcomes within 28 days in prespecified subgroups; results of multivariable logistic regression analysis. *Abbreviations* OR, odds ratios; CI, confidence interval; SpO_2_, oxygen saturation measured by pulse oximetry; BMI, body mass index. ^a^There was no three-way interaction (*P* = .72). Among the three possible two-way interactions, only the interaction “intervention by initial SpO_2_ < or ≥ 95%” was significant (*P* = .021) and was kept in the model. ^b^There was no three-way interaction (*P* > .99). Among the three possible two-way interactions, only the interaction “intervention by BMI < or ≥ 30 kg/m^2^” was significant (*P* = .049) and was kept in the model. ^c^There was no three-way interaction (*P* = .97). Among the three possible two-way interactions, only the interaction “intervention by initial SpO_2_ < or ≥ 95%” was significant (*P* = 0.02) and was kept in the model. ^d^There was no three-way interaction (*P* = .93). Among the three possible two-way interactions, only the interaction “intervention by initial SpO_2_ < or ≥ 95%” was significant (*P* = .01) and was kept in the model. ^e^There was no three-way interaction (*P* = .79). There was no significant interaction among the three possible two-way interactions (all *P* > .05). None of those interaction terms were introduced in the logistic model

Regarding the primary outcome, logistic regression showed a statistically significant interaction of the intervention with initial SpO_2_ (< or ≥ 95%) (*P* = 0.019), but not body mass index (< or ≥ 30 kg/m^2^) (*P* = 0.51). Patients with initial moderate oxygenation impairment (SpO_2_ < 95%) more frequently underwent NIV or invasive ventilation or died than patients with mild initial oxygenation impairment (SpO_2_ ≥ 95%) (21/43, 24.4% vs 15/92, 16.3%). The intervention had no statistically significant effect on the primary outcome in strata formed according to initial SpO_2_ or body mass index (Fig. 2). The risk of intubation, or intubation or death was lower in the prone position than in the usual care group for patients with initial SpO_2_ ≥ 95%: aOR 0.09, 95% CI 0.01–0.76, *P* = 0.040 and aOR 0.09, 95% CI 0.03–0.27, *P* = 0.027, respectively. For patients with body mass index < 30 kg/m^2^, the risk of NIV was increased with prone position versus usual care alone (aOR 9.85, 95% CI 1.29–75.00, *P* = 0.027).

Overall, similar results were found for the primary and secondary outcomes and for prespecified subgroups when excluding the three patients with do-not-intubate order after inclusion (data not shown).

### Per-protocol analysis

The per-protocol population comprised 42 (31.1%) patients of the intervention group who spent > 2 h in the prone position each day and the 131 (99.2%) patients of the usual care group who never laid prone. The treatment failure rate was 7.1% in the prone position group and 13.0% in the usual care group (aOR 0.71, 95% CI 0.18–2.77) (in Additional file 2, page 7). Secondary outcomes did not differ significantly between groups. The small size of the intervention group precluded subgroup analysis.

### Exploratory analyses

**Fig. 3:**
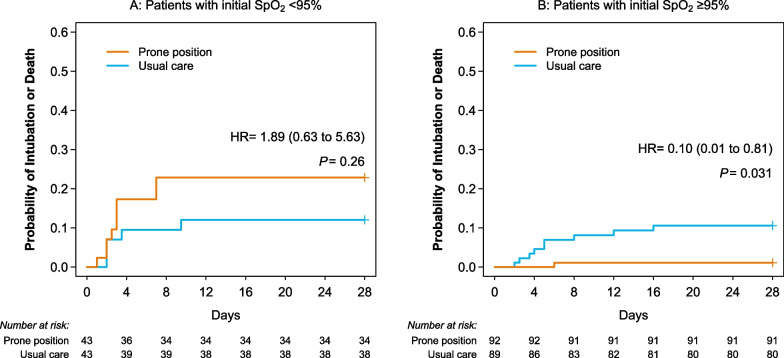
Probability of intubation or death using time-to-event analysis. *Abbreviations* HR, hazard ratio; SpO_2_, oxygen saturation measured by pulse oximetry. Note that the y axis is truncated at 0.6 to make the curves easily distinguishable. Groups were compared by Cox proportional hazards analysis (see Additional file 2, page 8)

Time-to-event analyses confirmed the results found by logistic regression in the whole study population and in prespecified subgroups (in Additional file 2, pages 9–16), i.e. no effect of the intervention on the primary outcome and a possible favourable effect of the intervention on rates of intubation, or intubation or death in the whole study population as well as in patients with the least oxygenation impairment [SpO_2_ ≥ 95% on inclusion], which contrasts with a possible unfavourable effect for patients with moderate hypoxemia (SpO_2_ < 95% on inclusion) (Fig. 3).

## Discussion

In this pragmatic RCT of COVID-19 patients requiring supplemental oxygen and hospitalised in medical wards, prone positioning did not significantly reduce the rate of NIV or intubation or death at 28 days as compared to usual care. Secondary analyses showed that the probability of intubation or death was lower in the prone position group, a trend driven by results for patients with mild oxygenation impairment on inclusion (SpO_2_ ≥ 95%).

Our findings concerning the global rates of intubation and death of COVID-19 patients initially hospitalised in medical wards are comparable to published results, ranging from 1 to 14% for intubation and 0–13% for death [12, 22, 28]. The observed rate of transfer to an ICU of about 15% was similar to those previously published (4.5–30%) but may be considered high given that we excluded patients at risk (high respiratory rate at screening, known severe respiratory comorbidity) [12, 28].

Our main result contrasts with results of previous studies. First studies of prone position in awake patients with COVID-19 outside the ICU showed that prone positioning was feasible and that a prone position session of 3.5 h may improve oxygenation [14, 17]. In a retrospective study in medical wards, the rate of upgrading the respiratory support at day 14 was lower for patients with prone positioning than the usual care group (31% vs 53%) [18]. In a large retrospective study of 827 non-intubated COVID-19 patients (in or outside the ICU), awake prone positioning was associated with reduced risk of intubation and mortality [29].

More recently, an RCT of 248 patients in medical wards yielded results similar to ours [22]. However, the study was stopped early for futility. The rate of a composite endpoint comprising escalation of oxygen flow, need for NIV or invasive ventilation, or death was 14% in the prone positioning and usual care groups, and the median cumulative time that patients of the intervention group spent on prone position within the first 72 h was 6 h [IQR, 1.5–12.8] and therefore slightly above that observed in our study.

We found a possible protective effect of prone positioning against the risk of intubation or death in patients with the least oxygenation impairment (initial SpO_2_ ≥ 95%) and observed an opposite trend in patients with moderate oxygenation impairment (initial SpO_2_ < 95%), the latter not statistically significant possibly because of insufficient statistical power. This is an unexpected finding because a large international meta-trial recently demonstrated that prone positioning protected critically ill COVID-19 patients with severe hypoxemia against intubation or death [7]. Additionally, a recent meta-analysis found no effect on intubation rate in patients treated with conventional oxygen therapy or outside the ICU [30]. Moreover, a recent Canadian RCT demonstrated no impact of initial hypoxemia level on the effect of prone positioning [22]. However, in this trial, the initial assessment of oxygenation was not standardised. In our study, the benefits of prone positioning by reducing lung stress and strain may have prevented the worsening of lung injury in patients with the least oxygenation impairment. Conversely, self-prone positioning with minimal external assistance may have been too oxygen-demanding to be tolerated by patients with more pronounced oxygenation impairment, thereby preventing any mechanical benefit of prone positioning.

Prone positioning in medical wards with low nurse-to-patient ratio requires more patient efforts than in the ICU. In our French hospital medical wards, nurses often have 12 (between 11 and 16 at night) patients to care for and receive help from one auxiliary nurse. This ratio is far below the nurse-to-patient ratio of 1:4 reported in the recent RCT conducted in medical wards in Canada [22] and could explain the lack of patient adherence and why time and duration of prone position session recordings were lacking for 14% of patients in our study.

Longer durations of daily prone position sessions likely benefit patients [31]. However, increasing patient adherence and tolerance in medical wards is a real challenge. One small RCT showed a very low patient adherence and concluded that prone positioning was not feasible in medical wards. Other trials failed to increase the patient adherence with smartphone-guided instructions or other means [19, 22, 28, 32]. Despite the use of a multifaceted intervention to increase patient adherence in the aforementioned Canadian trial, the time spent by patients in the prone position was rather similar to that in our study, which did not include specific procedures to motivate patients or additional staff. Given the difficulty of keeping patients awake in the prone position and our differing or even opposite results between patients with mild and moderate oxygenation impairment, perhaps prone positioning should be offered only to the least hypoxemic patients and those with the best tolerance. This situation would deserve sufficiently powered studies to be confirmed.

### Limitations

This study has several limitations. First, because of the nature of self-prone positioning, blinding patients and caregivers was not possible.

Second, subgroup analyses were underpowered.

Third, as for other studies [22, 33, 34], we studied a composite outcome comprising the need for NIV. Because the indication and criteria for initiation for NIV for patients with hypoxemic respiratory failure due to pneumonia are not well established, the triggers for its initiation may have differed across hospital/wards and among clinicians with different habits and skills, thereby introducing a possible centre effect. In this context, reasons for more frequent use of NIV in the prone position group were not clear. At the time of designing the study, while workload pressure and occupancy rate were maximal in the ICUs, we considered that NIV use in medical wards might represent a frank escalation of ventilatory support, perhaps replacing or deferring intubation in some patients in the context of shortage of ICU beds. In retrospect, perhaps considering only intubation and/or mortality as primary outcomes would have been a better option. Twenty patients underwent NIV within 28 days (12 in prone position group and 8 in usual care group). Of these, nine in the prone position group and three in the usual care group underwent NIV before transfer to the ICU, suggesting that the use of NIV actually was motivated by a true worsening of the patient’s respiratory status. The global rate of intubation or death within 28 days among patients who underwent NIV was 40% (8/20), but only 25% (3/12) in the prone position group compared with 62.5% (5/8) in the usual care group. Whether the use of NIV in some centres may have been more influenced by habits of clinicians than by true patients respiratory worsening and, on the other hand, whether the combination of prone positioning and NIV may have been protective against intubation or death are questions that cannot be answered by our study, which was neither designed nor sized for that purposes.

Fourth, criteria for intubation were not defined a priori and were at the discretion of wards or ICU physicians.

Fifth, the small proportion of patients initially treated with nasal high-flow oxygen (NHFO) therapy (4.5%) prevented any meaningful comparison between the different types of oxygen therapy. The choice we made not to include the use of NHFO therapy into our composite primary endpoint as an initial step of ventilatory support escalation is debatable. However, during the first surge of the COVID-19 pandemic, when we designed the study, the use of NHFO was not recommended due to the risk of aerosolisation of viral particles. After the first surge, we did not make any modification to the protocol, considering NHFO therapy to represent a far lesser therapeutic escalation than the use of NIV or intubation. Since then, several RCTs showed that NHFO did not significantly reduce ventilatory support escalation compared with standard oxygen in COVID-19 patients [35–37].

Sixth, the study design did not allow for an assessment of the impact of duration of prone position on outcomes because it was dependent on patient tolerance and different durations were not protocol-mandated.

Seventh, the variability in time from enrolment to prone positioning could be explained by the extra workload of caregivers during the pandemic. Caregivers had less time than usual to encourage and help patients to self-prone position. It is also possible that some patients were too tired or dyspneic for prone positioning after the enrolment.

Eighth, the night-time position was not recorded because of the extra workload of caregivers and could have impacted our results. The duration of prone position may have been determined by disease severity, which would bias any analysis towards worse outcomes associated with shorter prone position durations.

Ninth, whether patients of both groups have lied prone at night was not recorded. However, only 10% of patients declared prone position as their preferred sleeping position, and patients of the usual care group were clearly instructed not to lie prone at night. Therefore, we think unlikely that this could have biased our results. Finally, periods of prone position applied to patients of the intervention group were relatively short, which may explain our negative main result.

### Conclusions

Among COVID-19 patients hospitalised in medical wards and requiring supplemental oxygen, prone positioning did not reduce the risk of NIV or intubation or death. Secondary and prespecified subgroup analyses provide hypothesis for future research.

### Supplementary Information

**Additional file 1**. Protocol.

**Additional file 2**. Supplementary files.

## Data Availability

The datasets used and/or analysed during the current study are available from the corresponding author on reasonable request.

## Abbreviations

NHFO: Nasal high-flow oxygen
FiO_2_: Inspired fraction of oxygen
ICU: Intensive care unit
NIV: Non-invasive ventilation
RCT: Randomised clinical trial
WHO: World Health Organization
