# Supplementary material for: Prone position versus usual care in hypoxemic COVID-19 patients in medical wards: a randomised controlled trial
Source: Crit Care. 2023 Jun 17;27:240. doi: 10.1186/s13054-023-04529-z (PMC10276908; doi:10.1186/s13054-023-04529-z)
Supplement: Supplementary file 1 — Additional file 1. Protocol. [file 13054_2023_4529_MOESM1_ESM.docx]

**Clinical trial involving human participants, including only minimal risks and constraints (French Law reference: catégorie 2 du L1121-1)**

**PROVID-19**

Does awake prone positioning prevent the use of mechanical respiratory support or death in COVID-19 patients on standard oxygen therapy hospitalized in general wards? A multicenter randomized controlled trial: the PROVID-19 protocol

Most recent approved version the Ethics Committee: Version n°6.0– October 20, 2021

| Sponsor | | | | |
| --- | --- | --- | --- | --- |
| **Centre hospitalier régional d’Orléans / Regional hospital center of Orléans** Direction des affaires médicales et de la recherche / Research and Medical Affairs Department 14 avenue de l’hôpital  CS 86709 45067 Orléans Cedex 02  France | | | |  |
| COORDINATING INVESTIGATOR | | | | |
| **NAY Mai-Anh** Médecine Intensive Réanimation / Intensive Care Unit 14 avenue de l’hôpital CS 86 709 45067 Orléans Cedex 02 France | | | | 🕿 +33 2.38.57.52.53  [mai-anh.nay@chr-orleans.fr](mailto:mai-anh.nay@chr-orleans.fr) |
| Clinical trial protocol | | | | |
| **File number** | | CHRO-2020-09 | | |
| **N° IDRCB** | | **2020-A01151-38** | | |
| AUTHORIZATIONS | | | | |
| Independent Ethics Committee (Comité de Protection des Personnes) | | | | |
| CPP | File number:   \| CPP 1279 HPS2 \| \| --- \| | | Date:   \| First approved version: April 24, 2020 (version N°1.2)  Most recent version: October 20, 2021 (Version n°6.0) \| \| --- \| | |
| Competent authority (Agence Nationale de Securite du medicament et des produits de sante) | | | | |
| ANSM | Date of transmission of information:   \| April 24, 2020 \| \| --- \| | | | |
| French Data Protection Commission (CNIL France) | | | | |
| Declaration of Compliance to the baseline methodology (MR-001): March 10, 2017  This declaration is also applied to this protocol. | | | | |

Table of contents

[Consent form 6](#_Toc102172886)

[Summary 7](#_Toc102172887)

[1. General information 14](#_Toc102172888)

[1.1. Project Leaders 14](#_Toc102172889)

[1.2. Scientific Committee 15](#_Toc102172890)

[1.3. Supervisory board 15](#_Toc102172891)

[1.4. Collaborating centers 15](#_Toc102172892)

[2. Context and scientific justification 16](#_Toc102172893)

[2.1. Pathology description and results of avalaible clinical and non-clinical trials 16](#_Toc102172894)

[2.2. Description of the considered strategy 21](#_Toc102172895)

[3. Objectives of the study 21](#_Toc102172896)

[3.1. Primary objective 21](#_Toc102172897)

[3.2. Secondary objectives 21](#_Toc102172898)

[3.3. Primary endpoint 22](#_Toc102172899)

[3.4. Secondary endpoints 22](#_Toc102172900)

[4. Population 22](#_Toc102172901)

[4.1. Inclusion criteria 22](#_Toc102172902)

[4.2. Non-inclusion criteria 23](#_Toc102172903)

[4.3. Recruitment procedure 23](#_Toc102172904)

[4.4. Risk/Benefit 24](#_Toc102172905)

[5. Research design 25](#_Toc102172906)

[5.1. Method 25](#_Toc102172907)

[5.2. Research-Specific Procedures 26](#_Toc102172908)

[5.3. Trial progress 28](#_Toc102172909)

[5.4. Research duration 29](#_Toc102172910)

[5.5. Measures to reduce and avoid bias 30](#_Toc102172911)

[5.6. Data input in the case report form used as source data 30](#_Toc102172912)

[5.7. Rules for definitive or temporary termination 31](#_Toc102172913)

[6. Drugs administered 32](#_Toc102172914)

[6.1. Authorized treatments 32](#_Toc102172915)

[6.2. Unauthorized treatments 32](#_Toc102172916)

[6.3. Emergency treatment 32](#_Toc102172917)

[7. Safety assessment 32](#_Toc102172918)

[7.1. Definitions 32](#_Toc102172919)

[7.2. Adverse event reporting 33](#_Toc102172920)

[8. Statistic 35](#_Toc102172921)

[8.1. Statistical methods 35](#_Toc102172922)

[8.2. Number of participants to be included in the research 36](#_Toc102172923)

[8.3. Considered degree of statistical significance 36](#_Toc102172924)

[8.4. Statistical research termination criterion 36](#_Toc102172925)

[8.5. Management of missing, unused or invalid data 36](#_Toc102172926)

[8.6. Selection of participants to be included in the analysis 36](#_Toc102172927)

[9. Data and source document access rights 37](#_Toc102172928)

[9.1. Data access rights 37](#_Toc102172929)

[9.2. Source documents 37](#_Toc102172930)

[9.3. Data confidentiality 37](#_Toc102172931)

[10. Quality control and assurance 38](#_Toc102172932)

[11. Ethical considerations 39](#_Toc102172933)

[11.1. Independent Ethics Committee 39](#_Toc102172934)

[11.2. Substantial amendments 39](#_Toc102172935)

[11.3. Patient information and consent form 39](#_Toc102172936)

[11.4. Definition of the exclusion period 40](#_Toc102172937)

[11.5. Compensation 40](#_Toc102172938)

[11.6. Registration of persons consenting to biomedical research on the national data file 40](#_Toc102172939)

[12. Data and document use and storage 40](#_Toc102172940)

[12.1. Case report form (CRF) 40](#_Toc102172941)

[12.2. Data input and data production 40](#_Toc102172942)

[12.3. French data protection commission (CNIL-France) 40](#_Toc102172943)

[12.4. Archiving 41](#_Toc102172944)

[13. Funding and insurance 41](#_Toc102172945)

[13.1. Budget 41](#_Toc102172946)

[13.2. Insurance 42](#_Toc102172947)

[14. Practical aspects of the study 42](#_Toc102172948)

[15. Rules relating to publication 42](#_Toc102172949)

[16. Sources 44](#_Toc102172950)

[17. Appendices 47](#_Toc102172951)

# Consent form

INVESTIGATOR SIGNATURE

| I have read all the pages of the protocol for the clinical trial sponsored by Orleans Regional Hospital. I confirm that it contains all the information required to conduct the trial.  I therefore undertake to conduct this trial according to the protocol and to the terms and conditions set out in this protocol.  I undertake to conduct this trial in accordance with:   - the principles of the Helsinki Declaration - international GCP rules and guidelines (ICH-E6) but also the French version (règles de bonnes pratiques cliniques pour les recherches biomédicales portant sur des médicaments à usage humain - décisions du 24 novembre 2006) - national legislation and regulations relating to clinical trials, - the European directive on clinical trials [2001/20/EC].   I also undertake to provide the investigators and the qualified members of my team with access to copies of this protocol and to the trial documents in order to act in full compliance with the provisions set out in these documents. |
| --- |
| **NAME:**  Signature: Date:  *In accordance with the provisions of the law relating to data processing, files and freedoms, you have at any time a right of access and of correction of the computerized data concerning you (law n° 2004-801 of August 6, 2004 modifying the law n° 78-17 of January 6, 1978 relating to data processing, files and freedoms).* |

**SPONSOR SIGNATURE**

| **Sponsor:** | |
| --- | --- |
| NOM: Antoine LEBRERE  Research Department Director  Signature: | Date: ___________________ |

# Summary

| TiTLE | **Provid-19**  **Does awake prone positioning prevent the use of mechanical respiratory support or death in COVID-19 patients on standard oxygen therapy hospitalized in general wards? A multicenter randomized controlled trial: the PROVID-19 protocol.** |
| --- | --- |
| SPONSOR | **Centre hospitalier régional d’Orléans / Orleans Regional Hospital** Direction des affaires médicales et de la recherche / Research and Medical Affairs Department 14 avenue de l’hôpital  45067 Orléans Cedex 02 France |
| Investigator, Project Coordinator | **NAY Mai-Anh**  **Centre hospitalier régional d’Orléans / Orleans Regional Hospital** Service de Médecine Intensive et Réanimation / Intensive Care unit 14 avenue de l’hôpital  45067 Orléans Cedex 02 France |
| Protocol version | Most recent approved version by the Ethics Committee: n°6.0 dated October 20, 2021 |
| Project Justification / context | **RATIONAL FOR COVID-19:**  The current global SARS-Cov-2 viral pandemic, which is responsible for a new infectious disease called COVID-19, is a major health concern. The mortality of hospitalized patients with COVID-19 is 5% to 15% (MURTHY, JAMA, 2020). In 15% to 40% of cases, the disease is responsible for respiratory complications, and the most serious is acute respiratory distress syndrome (ARDS) (HUANG, LANCET 2020; ZHOU, LANCET 2020; WU, JAMA INTERN MED 2020).  The access to an intensive care unit (ICU) is about 15% to 30%, with mechanical ventilation use 10% to 25% from the first Chinese studies (HUANG, LANCET 2020; GUAN, N ENGL J MED 2020, GUO, LANCET CARDIOLOGY 2020, WANG, JAMA 2020). ARDS is present in two thirds of cases (WANG, JAMA 2020). Mortality of patients with ARDS is high (53%) (WU, JAMA INTERN MED 2020). In a smaller population of 21 Americans admitted to the ICU, 70% required invasive mechanical ventilation (ARENTZ, JAMA 2020).  The management of hospitalized COVID-19 cases is essentially symptomatic, with oxygen support in mild forms and invasive mechanical ventilation in the most severe forms. Drug studies are under way to find out how to avoid the worsening of respiratory symptoms.  The seriousness of this pandemic is related to the saturation of ICUs (GRASSELLI, JAMA 2020). Thus, trying to prevent worsening as much as possible could reduce the need for invasive or non-invasive mechanical ventilation.  **RATIONALE FOR PRONE POSITIONING FOR ARDS:**  ARDS is defined by the Berlin definition (ARDS DEFINITION TASK FORCE, JAMA, 2012) as diffuse lung injury occurring in patients with a predisposing risk factor.  Prone positioning (PP) has been shown to decrease mortality in patients with moderate to severe ARDS in the ICU.  In a study by Ding et al (DING, CRIT CARE, 2020), in non-intubated patients, the combination of PP (2 hr twice a day) and high flow oxygen therapy decreased the need for intubation in patients with moderate to severe ARDS.  We hypothesize that the use of PP in patients with spontaneous breathing (under conventional oxygen therapy) during acute respiratory distress caused by SARS-Cov-2 infection (COVID-19) would result in decreased incidence of intubation or non-invasive ventilation at 2 pressure levels or death as compared with usual care. |
| Primary Objective | To demonstrate that PP in spontaneously breathing patients decreases the risk of acquiring the following events, which can be seen as a composite endpoint:  - Endotracheal intubation  - Or non-invasive ventilation at 2 pressure levels  - Or death |
| Secondary objectives | To demonstrate that the use of PP improves (decreases) the World Health Organization (WHO) ordinal scale score by 2 points from randomization (CAO; N Engl J Med; 2020)  To demonstrate that PP in spontaneously breathing patients reduces the need for tracheal intubation and invasive mechanical ventilation.  To demonstrate that PP in spontaneously breathing patients reduces the use of non-invasive ventilation at 2 pressure levels.  To demonstrate that PP in spontaneously breathing patients reduces the time on oxygen therapy.  To demonstrate that PP reduces the length of hospital stay  To demonstrate that PP in spontaneously breathing patients decreases mortality at day 28 in COVID-19 patients.  To compare the in-hospital mortality of the 2 groups (PP and usual care)  To compare the incidence of the need for transfer to ICU between the 2 groups  To compare the incidence of the use of non-invasive ventilation and intubation between the 2 groups, over the entire hospital stay when the stay is longer than 28 days. |
| Primary Endpoint | The rate of treatment failure defined as a composite endpoint comprising intubation and/or non-invasive ventilation at 2 pressure levels and/or death, in each group. |
| Secondary Endpoints | The duration (in days) to improve (score decrease) the clinical WHO ordinal scale score from randomization to 28 days between the 2 groups  The rate of endotracheal intubation (%) for invasive mechanical ventilation at 28 days between the 2 groups  The rate of non-invasive ventilation at 2 pressure levels at 28 days between the 2 groups  The time on oxygen therapy from inclusion to day 28 between the 2 groups  The hospital length of stay between the 2 groups  The mortality at 28 days and during the hospital stay between the 2 groups  The rate of transfer to ICU at 28 days  The rate of use of non-invasive ventilation at 2 pressure levels and intubation over the entire hospital stay. |
| Study Method/ Design | This will be 2 parallel-group randomized controlled trial with 1:1 allocation ratio to usual care or intervention (PP).  The control group will be usual care in all participating departments/hospitals. The prone position will not be allowed during the day (allowed at night if it is the patient’s natural sleeping position).  The intervention group will undergo the following:   - Each day of hospitalization, at least 2 PP sessions during the day, with the objective to spend as much time as possible in PP if tolerated and a minimum of 2.5 hr cumulative time in PP during the day. - A session of PP as long as possible at night. Patients will be encouraged to sleep in PP as much as possible at night, knowing that monitoring the number of hours will not be feasible in COVID-19 medical wards in the current context.   Bedridden patients who are not able to self-position to the prone position or with minimal assistance of one person will not be included in the study.  Barriers will be positioned to avoid a potential fall out of bed.  The patient will be allowed to choose the best PP as long as the back of the thorax is not compressed by the bed. |
| Inclusion criteria | Patients > 18 and < 85 years old  Patients hospitalized with a laboratory-confirmed SARS-CoV-2 infection  Patients on oxygen therapy (nasal cannula, mask or high-flow nasal oxygen therapy)  Patients able to self-position to the prone position or with minimal assistance of one person  Signed the consent form  Patients hospitalized in a COVID-19 medical ward for < 72 hr |
| Non-inclusion criteria | Patients on oxygen therapy or continuous positive airway pressure or non-invasive ventilation at home  Patients with chronic obstructive pulmonary disease (COPD) (Gold stage 3 or 4)  Patients with known chronic diffuse interstitial lung disease  Patients with a chronic neuromuscular disease  Contraindication to PP (recent thoracic trauma, pneumothorax, unstable spine or pelvis fractures etc.)  Deep vein thrombosis or pulmonary embolism with curative anticoagulation for < 48 hr  Hemodynamic instability (mean arterial pressure < 65 mm Hg) persisting for > 1 hr  Respiratory rate > 40 cycles/min.  Excessive use of accessory respiratory muscles (as determined by the clinician)  Indication for curative non-invasive ventilation (acute pulmonary edema or acute hypercapnic respiratory failure)  Intestinal occlusive syndrome  Patients unable to protect upper airways  Patients discharged from intensive care after treatment with invasive or non-invasive ventilation for COVID-19  Inability to understand French or to follow instructions to perform awake PP.  Patients not affiliated with or excluded from the French social security system or under law protection (namely minors, pregnant or breastfeeding women, persons deprived of their liberty by court or administrative decision)  Do not intubate order |
| Strategy / procedures | - **Prone position plus usual care** - **Usual care alone** |
| Number of Patients | 268 |
| Duration of investigation | Inclusion period: Epidemic period of COVID-19  **Participation time for each patient.** The study intervention will stop when one of the following events is reached: initiation of non-invasive or invasive ventilation, death, or still hospitalized at day 28 without event. Patients hospitalized for > 28 days will continue to be followed in the study (rate of use of non-invasive or invasive ventilation, rate of admission to an ICU, death).  The duration of participation for each patient corresponds to the duration of the hospitalization (all wards combined) or longer in case of severe adverse events that would require longer follow-up. |
| Expected impacts | In the event that the PP would reduce the rate of non-invasive or invasive ventilation or death, it could be proposed as a standard-of-care technique for hypoxemic COVID-19 patients. |

List of acronyms

| AE  ARDS  CRA  CRF | adverse event  acute respiratory distress syndrome  clinical research assistant  case report form |
| --- | --- |
| COPD | chronic obstructive pulmonary disease |
| GCP | Good Clinical Practice |
| PP | prone position |

# General information

## Project Leaders

| Name of the clinical trial:  **PROVID-19: Does awake prone positioning prevent the use of mechanical respiratory support or death in COVID-19 patients on standard oxygen therapy hospitalized in general wards? A multicenter randomized controlled trial: the PROVID-19 protocol** | |
| --- | --- |
| **Sponsor:** | CHR D’Orléans / Orléans Regional Hospital  Direction des affaires médicales et de la recherche / Research and Medical affairs Department  14 avenue de l’hôpital CS 86709 45067 Orléans Cedex 02  France |
| **Coordinating Investigator:** | Dr NAY Mai-Anh, MD  Médecine Intensive Réanimation / Intensive Care Unit Department  14 avenue de l’hôpital  CS 86709 45067 Orléans Cedex 02  France |
| **Scientists:** | Dr BOULAIN Thierry, MD  Médecine Intensive Réanimation / Intensive Care Unit Department  14 avenue de l’hôpital  CS 86709 45067 Orléans Cedex 02  France  FOSSAT Guillaume, PT  Médecine Intensive Réanimation / Intensive Care Unit Department  14 avenue de l’hôpital  CS 86709 45067 Orléans Cedex 02  France |
| **Pharmacovigilance:** | NA |
| **Statistics:** | Dr BOULAIN Thierry, MD  Médecine Intensive Réanimation / Intensive Care Unit Department  14 avenue de l’hôpital  CS 86709 45067 Orléans Cedex 02  France |

## Scientific Committee

Fossat Guillaume, PT, Médecine Intensive Réanimation/Intensive Care Unit Department, CHR d’Orléans

Nay Mai-Anh, MD, Médecine Intensive Réanimation/Intensive Care Unit Department, CHR d’Orléans

Boulain Thierry, MD, Médecine Intensive Réanimation/ Intensive Care Unit Department, CHR d’Orléans

Bigot Adrien, MD, Médecine Interne et Immunologie Clinique/ Department of Internal Medicine, CHRU de Tours

## Supervisory board

NA

## Collaborating centers

| **Center No.** | **Department** | **Hospital Center** | **Principal Investigator** |
| --- | --- | --- | --- |
| 001 | Pneumology | CHR Orléans | Dr DRUELLE Sylvie |
| 002 | Infectious and Tropical Diseases | CHR Orléans | Dr SEVE Aymeric |
| 003 | Pneumology | CHRU de Tours | Pr PLANTIER Laurent |
| 004 | Internal Medicine | CHRU de Tours | Pr MAILLOT François |
| 005 | Infectious Diseases | CHRU de Tours | Pr BERNARD Louis |
| 006 | Infectious Diseases | CH de Dax | Dr NYAMANKOLLY Elsa |
| 007 | Infectious Diseases | CH de La Rochelle | Dr POUGET-ABADIE Xavier |
| 008 | Diabetes, Endocrinology and Nutrition | APHP Lariboisière | Pr GAUTIER Jean-François |
| 009 | Infectious Diseases | APHP Lariboisière | Pr SELLIER Pierre-Olivier |
| 010 | Internal Medicine | APHP Lariboisière | Dr LOPES Amanda |
| 011 | Infectious Diseases | CH Vendée | Dr MORRIER Marine |
| 012 | Infectious Diseases | CH de Perpignan | Dr COLOMBAIN Léa |
| 013 | Pneumology | CH de Perpignan | Dr COLOMBAIN Léa |
| 014 | Pneumology | CHI de Cornouaille | Dr BIZIEN Nicolas |
| 015 | Pneumology | HEGP | Dr PLANQUETTE Benjamin |
| 016 | Internal Medicine | CH de Mont de Marsan | Dr LACASSIN-BELLER Flore |
| 017 | Internal Medicine and General Medicine | CH de Blois | Dr CLEMENT Jérémy |
| 018 | Infectious Diseases | CH Le Mans | Dr LAINE Jean-Baptiste |
| 019 | Pneumology | CH Princesse Grace (Monaco) | Dr PERRIN Christophe |
| 020 | Pneumology and General Medicine | CH Breatagne Atlantique | Dr CORVAISIER Grégory |

# Context and scientific justification

## Pathology description and results of available clinical and non-clinical trials

### COVID-19

The current global SARS-Cov-2 viral pandemic, which is responsible for a new infectious disease called COVID-19, is a major health concern. The mortality of hospitalized patients with COVID-19 is 5% to 15% (MURTHY, JAMA, 2020). In 15% to 40% of cases, COVID-19 is responsible for respiratory complications, among which the most serious is acute respiratory distress syndrome (ARDS) (HUANG, LANCET 2020; ZHOU, LANCET 2020; WU, JAMA INTERN MED 2020).

The access to an intensive care unit (ICU) is about 15% to 30%, with mechanical ventilation use 10% to 25% from the first Chinese studies (HUANG, LANCET 2020; GUAN, N ENGL J MED 2020, GUO, LANCET CARDIOLOGY 2020, WANG, JAMA 2020). ARDS is present in two thirds of cases (WANG, JAMA 2020). The mortality of patients with ARDS is high (53%) (WU, JAMA INTERN MED 2020). In a smaller population of 21 Americans admitted to an ICU, 70% required invasive mechanical ventilation (ARENTZ, JAMA 2020).

The management of hospitalized COVID-19 cases is essentially symptomatic, with oxygen support in mild forms and invasive mechanical ventilation in the most severe forms. Drug studies are under way to find out how to avoid the worsening of respiratory symptoms.

The seriousness of this pandemic is related to the saturation of ICUs (GRASSELLI, JAMA 2020). Thus, trying to prevent worsening as much as possible could reduce the need for invasive or non-invasive mechanical ventilation.

### Acute respiratory distress syndrome (ARDS)

ARDS is a diffuse pulmonary inflammation. Various diseases can be responsible for ARDS, exhibiting acute generalized inflammation with significant cytokine discharge and diffuse damage to the vascular endothelium. ARDS is defined by the Berlin definition (ARDS DEFINITION TASK FORCE; JAMA; 2012) as diffuse lung injury occurring in patients with a predisposing risk factor. The criteria are as follows:

- Occurrence within 1 week of known insult or new or worsening respiratory symptoms

- Bilateral opacities not fully explained by effusions, lobar or lung collapse, or nodules on imaging

- Diagnosis of respiratory failure not fully explained by heart failure or fluid overload

- Presence of hypoxemia according to the PaO2/FiO2 ratio (P/F) with a positive end-expiratory pressure (PEEP) ≥ 5 cmH2O.

This last condition leads to classification into 3 ARDS subgroups:

o P/F 200 to 300: mild ARDS

o P/F 200 to 100: moderate ARDS

o P/F < 100: severe ARDS

Currently, excluding the SARS-CoV-2 epidemic, 5% of patients hospitalized with mechanical ventilation have ARDS criteria.

The main risk factors for ARDS are pneumonia, sepsis, inhalation of gastric fluid, chest trauma, pancreatitis and drowning.

Chest CT scan is an aid to diagnosis; it can detect healthy areas (properly ventilated) from unventilated areas (ground glass opacities or consolidation). Chest ultrasonography and the presence of B-lines can also identify areas affected by ARDS.

ARDS evolves in the following 3 phases that are theoretically successive but that can be mixed depending on mechanical ventilation and the occurrence of nosocomial infection (ALIAGA; Intensive Care Med; 2015):

1. Exudative or inflammatory phase

2. Proliferation phase (type II alveolar cells, fibroblasts and respiratory breakdown with increased risk of mortality)

3. Fibrotic phase (major increase in mortality risk, with a reduction in lung compliance)

The understanding of the physiology of ARDS has led to an improvement in ventilatory management. Ventilation must be "protective" (use of low insufflated volumes) and ensure adequate oxygenation with sufficient CO_2_ clearance. It allows for decreasing the extent of lung damage produced by the evolution of ARDS (ventilator-induced lung injuries).

Pharmacological and non-pharmacological strategies for the management of ARDS are outlined in the table below (UMBRELLO; Int J Mol Sci; 2016):


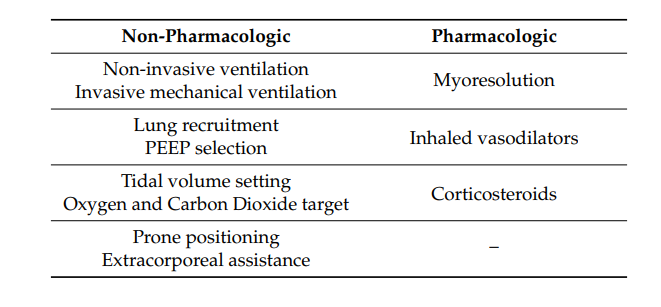


Prone positioning (PP) could reduce mortality in ARDS patients. PP results in a more homogeneous distribution of pulmonary stress and strain, thus protecting the lung against ventilator-induced lung injuries. It also improves oxygenation with an increase of the P/F ratio, an increase of pulmonary perfusion ventilation ratio, and a decrease of PaCO_2_ and improves the ventilation in dorsal regions. PP should be used in all patients under mechanical ventilation with severe ARDS for 16 consecutive hours to improve mortality and success of weaning from mechanical ventilation (GUERIN ; N Engl J Med ; 2013).

However, few studies have evaluated PP in spontaneously breathing patients.

### ARDS and positioning

Different techniques exist for positioning patients with ARDS

In 2005, Hoste et al. (HOSTE; Intensive Care Med; 2005) showed that positioning patients with ARDS at 40° to 45° of uprightness in dorsal decubitus position improved the P/F ratio in all patients. This improvement was considered significant in 66% of patients. Patients who did not respond to this position had thoracic trauma or lung surgery. In this study, patients were so positioned for 12 hr, during which none had any major hemodynamic problems. Only 5 patients required vascular filling without the use of amines (511 ml). At the beginning of the positioning maneuver, 72.2% of the patients were on norepinephrine (0.44 μg/kg/min) and 22% on dobutamine (10 µg/kg/min). During the 12 hr of the technique, there was no significant change in hemodynamic parameters.

In 2006, Richard et al. (RICHARD; Intensive Care Med; 2006) showed that a pseudo-upright position (head of the bed raised to 45° and foot of the bed lowered to 45°) significantly improved the PaO_2_ (94 ± 33 up to 142 ± 49 mmHg) in some responder patients (11 of 16). The end-expiratory lung volume was also increased in the "pseudo-upright position". The physiological explanation would be alveolar recruitment resulting in a greater lung volume without modification of the thoraco-pulmonary viscoelastic properties or the thoraco-pulmonary specific compliance.

However, this pseudo-verticalization technique has not been studied further in ARDS. Indeed, the use of PP has become routine for managing ARDS in patients. A multicenter study by Guérin et al. (PROSEVA study), showed decreased mortality in > 400 ARDS patients. Daily use of PP for 16 hr reduced mortality by 50% on days 28 and 90 as compared with conventional management (GUERIN; N Engl J Med; 2013).

In 2011, in a prospective randomized cross-over study (ROBAK; Crit Care; 2011), combining prone and upright positions improved oxygenation in ARDS patients. The patient was placed in the prone position with the top of the bed raised and the foot of the bed lowered. The upright position corresponds to an elevation of the top of the bed and a lowering of the leg section, thus resulting in a tilting of the bed in a pro-trendy position. This position improved PP effects in responders. Non-responders had higher P/F ratio than responders at the time of PP [169 (13-257) vs 123 (90-149)]. Responders showed decreased oxygenation when the patient was repositioned in conventional PP. As soon as the patient was repositioned in the upright position, oxygenation again improved. This observation excluded a time-dependent effect on oxygenation. In this study, the measurement of effects on oxygenation was performed only over an 8-hr period.

In 2013, Dellamonica et al. (DELLAMONICA; Intensive Care Med; 2013) showed that ARDS patients responding to the upright position had lower lung compliance than non-responders [30 ml/cmH2O (22-38) vs 42 ml/cmH2O].

In spontaneously breathing patients, Ding et al. (DING, CRIT CARE, 2020) showed that the use of PP in non-intubated patients (2 hr twice a day) with high-flow oxygen therapy support decreased the need for intubation in patients with moderate to severe ARDS.

The prone position is not feasible for all patients with ARDS. The absolute contraindications are pregnancy, major hemodynamic instability, open abdominal surgery, and unstable fractures (GATTINONI; Am J Respir Crit Care Med; 2013).

### COVID-19, ARDS and PP

No studies have been published regarding the value of PP in non-intubated COVID-19 patients (whether classified as having "ARDS" based on their PaO_2_/FiO_2_ ratio assessed on spontaneous breathing [PaO_2_/FiO_2_<300 or <200] or not).

Two clinical observations result from published cohort studies of COVID-19 patients:

1) On admission to the hospital and ICU, chest CT most often shows a major interstitial syndrome with no or little alveolar filling or consolidation (ZHOU, AJR Am J Roentgenol, 2020).

2) Yet, daily observation of intubated COVID-19 patients shows that PP, which is known to result in a good response in terms of oxygenation only when there is alveolar filling of the declivities in the supine position, shows that a large majority of patients are responders.

To explain this "paradoxical" effect, the classic "re-aeration" of the filled or condensed declivities (which allows for readjustment of the ventilation/perfusion ratio towards a better ventilation) due to PP is not sufficient. The PP in this case may have a predominant effect on vascularization: a predominant effect on the "perfusion" part of the ventilation/perfusion ratio, which would lead to a better perfusion of the best ventilated areas and thus a decrease in the shunt effect. However, the best ventilated areas are difficult to localize and are likely to be diffuse.

COVID-19 coronavirus (2019-nCoV) enters the cell by binding the S1 part of its Spike protein to the angiotensin converting enzyme II (ACE2) receptor [ZHOU, NATURE, 2020]. The intensity of viral respiratory infection depends on the degree of host cell maturation and the level of expression of ACE2 and its receptor in cells of the respiratory epithelium and vascular endothelium [IA; J Virol; 2005]. ACE converts angiotensin I to angiotensin II, whereas ACE2 cleaves a different hexapeptide, angiotensin-1Y7 (Ang-1Y7) from angiotensin II. ACE2 regulates the renin-angiotensin system directly by reducing the coupling of angiotensin to the ACE receptor but also by activating a counter-regulatory pathway through the Ang 1-7/Mas receptor pathway [KHAN; Crit Care; 2017/JUILLERAT-JEANNERET; J Med Chem; 2020]. ACE inhibition (by classical ACE inhibitors) seems to have a protective effect in ARDS, without knowing whether it is the reduced ACE activity level or on the contrary the ACE/ACE2 balance more favorable to ACE2 that explains the protective power [LI; Schock; 2015.]

Angiotensin II is a potent vasoconstrictor, active on vascular smooth muscle cells. Ang(1-7) produced by ACE2 from angiotensin II induces arachidonic acid production and nitric oxide synthase activation in all tissues but predominantly in the brain [JUILLERAT-JEANNERET; J Med Chem; 2020.]. Thus, the ACE2 pathway has overall tissue-protective effects, notably tested in ARDS [KHAN; Crit Care; 2017], owing to its various anti-proliferative, anti-apoptotic, anti-fibrotic, and microvascular vasodilatation effects.

Therefore, the hypoxemia of COVID-19 may at least in part be related to a disruption of the ACE2 pathway at the pulmonary microvascular level owing to the congestion of its membrane receptors by viral proteins. In parallel, the same hypotheses, this time with the prospect of a pharmacological intervention on this pathway, have been put forward recently [Vaduganathan; N Engl J Med; 2020].

Of course, this scenario does not provide a clear explanation for the finding of a significant effect of PP on oxygenation in COVID-19 patients, but the changes in blood flow at the thoraco-pulmonary level brought about by the PP maneuver may be able to reduce, for an undetermined time, the microvascular detrimental effects of the partial inhibition of the ACE2 pathway. An effect, even if limited in time, would have a favorable effect on oxygenation, which could benefit the patient.

## Description of the considered strategy

We hypothesize that the use of prone position in spontaneously breathing COVID-19 patients under oxygen therapy would reduce the rate of intubation or non-invasive ventilation at 2 pressure levels.

# Objectives of the study

## Primary objective

To demonstrate that PP in spontaneously breathing patients decreases the risk of acquiring the following events, which can be seen as a composite endpoint:

- Endotracheal intubation

- Or non-invasive ventilation at two pressure levels

- Or death

However, we plan to include only patients without an order to not intubate so that if an imminent life-threatening respiratory occurs, intubation will be performed. Thus, only sudden unexpected death not likely to be related to respiratory function could occur and be counted in this composite criterion without endotracheal intubation or non-invasive ventilation.

## Secondary objectives

The duration (in days) to improve (score decrease) the clinical WHO ordinal scale score from randomization to 28 days between the 2 groups (PP and usual care)

The rate of endotracheal intubation (%) for invasive mechanical ventilation at 28 days between the 2 groups

The rate of non-invasive ventilation at 2 pressure levels at 28 days between the 2 groups

The time on oxygen therapy from inclusion to day 28 between the 2 groups

The hospital length of stay between the 2 groups

The mortality at 28 days and during the hospital stay between the 2 groups

The rate of transfer to an ICU at 28 days

The rate of use of non-invasive ventilation at 2 pressure levels and intubation over the entire hospital stay

## Primary endpoint

The rate of treatment failure defined as a composite endpoint comprising intubation and/or non-invasive ventilation at 2 pressure levels and/or death, in each group (PP and usual care).

## Secondary endpoints

The duration (in days) to improve the clinical WHO ordinal scale from randomization to 28 days between the 2 groups (PP and usual care). This scale is recommended by the WHO (<http://www.who.int/blueprint/priority-diseases/key-action/novel-coronavirus/en/>) and was used in clinical trial of COVID-19 (CAO; N Engl J Med; 2020). This scale is relevant but it could be difficult to use for overloaded wards during an epidemic to exhaustively survey all points. Moreover, because the unit of time is the day, the score is imprecise (e.g., for reasons of feasibility, it is not possible to envisage twice-daily surveying). For this reason, the scientific committee chose not to use this endpoint as primary endpoint.

The rate of endotracheal intubation (%) for invasive mechanical ventilation at 28 days between the 2 groups

The rate of non-invasive ventilation at two pressure levels at 28 days between the 2 groups

The time passed on oxygen therapy from inclusion to day 28 between the 2 groups

The hospital length of stay between the 2 groups

The mortality at 28 days and during the hospital stay between the 2 groups

The rate of transfer to ICU at 28 days

The rate of use of non-invasive ventilation at 2 levels pressure and intubation over the entire hospital stay.

# Population

## Inclusion criteria

Patients > 18 and < 85 years old

Patients hospitalized with a laboratory-confirmed SARS-CoV-2 infection

Patients on oxygen therapy (nasal cannula, mask or high-flow nasal oxygen therapy)

Patients able to self-position to the prone position or with minimal assistance of one person

Signed the consent form

Patients hospitalized in COVID-19 medical ward for < 72 hr

## Non-inclusion criteria

Patients on oxygen therapy or continuous positive airway pressure or non-invasive ventilation at home

Patients with COPD (Gold stage 3 or 4)

Patients with known chronic diffuse interstitial lung disease

Patients with a chronic neuromuscular pathology

Contraindication to prone position (recent thoracic trauma, pneumothorax, unstable spine or pelvis fractures etc.)

Deep vein thrombosis or pulmonary embolism with curative anticoagulation for < 48 hr

Hemodynamic instability (mean arterial pressure < 65 mm Hg) persisting for > 1 hr

Respiratory rate > 40 cycles/min

Excessive use of accessory respiratory muscles (as determined by the clinician)

Indication for curative non-invasive ventilation (acute pulmonary edema or acute hypercapnic respiratory failure)

Intestinal occlusive syndrome

Patients unable to protect upper airway

Patients discharged from intensive care after having treatment with invasive or non-invasive ventilation for COVID-19

Inability to understand French or to follow instructions to perform awake PP

Patients not affiliated or excluded from the French social security system or under law protection (namely minors, pregnant or breastfeeding women, persons deprived of their liberty by court or administrative decision)

Do not intubate order

## Recruitment procedure

Patients will be recruited in the "COVID" departments (excluding ICUs) of the hospitals participating in the study.

Participation in the study will be possible as soon as the patient presents a SARS-CoV-2 infection and is hospitalized in a medical ward dedicated to COVID-19 patients.

The information and written consent of the patients will be obtained on admission to the ward, and a copy of the information note and consent form will be given to the physician or physiotherapist personally.

The patient will be able to decline to participate in the study and the treatment will not be modified.

The patient will be free to decline the use of the data collected during the intervention and the care provided will not be modified.

## Risk/benefit

### Benefits of prone position during spontaneous breathing

Ding et al. (DING, Crit Care, 2020) showed that PP could increase the P/F ratio by 25 to 35 points as compared with before PP. Only 2 patients did not tolerate the use of PP during spontaneous breathing. PP does not require the use of sedation to tolerate this position. In a letter to the editor, Pérez-Nieto et al. reported that in a multicenter retrospective series of 6 patients admitted to the ICU for severe ARDS of non-infectious etiology, the use of PP in non-intubated patients would avoid the need for intubation in 3 of 6 cases. Obviously, these results do not allow us to conclude on the utility of PP but rather indicate the potential of this technique in non-intubated patient (PEREZ-NIETO, Crit Care, 2020).

### Drawback of PP during spontaneous breathing

The mean duration on PP was 2 hr in spontaneously breathing patients in the study by Ding et al. (DING; Crit Care; 2020). For our study, to obtain an optimal benefit, we propose a minimum of two PP sessions during the day with a minimum cumulative time of 2.5 hr.

### Individual benefits

This study is a prospective randomized controlled interventional study.

To date, there is no recommendation for the use of PP in non-critical COVID-19 patients. The benefits are uncertain in these patients, and we believe that the use of this technique would decrease (as it seems to do for other patients with moderate ARDS) the time on oxygen therapy and the use of invasive or non-invasive ventilation and thus decrease the need for intensive care.

### Collective benefits

Improvement of oxygenation for COVID-19 patients resulting in reduced hospital stays, reduced congestion in ICUs and reduced mortality.

### Risks

One of the complications of COVID-19 is the evolution of severe ARDS.

The risk of PP is pressure on the face and knees, thorax and abdomen. These risks were observed in only sedated patients with neuromuscular blockades under invasive mechanical ventilation with PP for 16 hr. In spontaneously breathing, aware patients, patients will be able to move to avoid excess pressure. We can assume that the patients are ambulant and are able to adapt their positions according to their feelings.

In the PP studies in awake patients, no adverse events (AEs) have been reported. However, the following events may require a return to standard position: feeling of respiratory discomfort or any other uncomfortable feeling for the patient.

All these events will be only considered serious if they lead to worsening of the health condition that was present prior to PP.

The usual monitoring will be applied by the medical and paramedical teams of the enrolling wards. The teams will be free to determine whether the situation requires a change on supine position.

### Strategies to minimize risks

If an AE occurs, the patient would return to the supine position and semi-sitting position immediately. We will record the reasons for ending the procedure.

If an AE occurs during the prone procedure, we would follow the procedure described in Chapter 7.

# Research design

## Method

The control group will receive the usual care in each department involved in the study. The semi-sitting position in the bed or the chair is the usual care used in current practice for positioning.

In addition to usual care, the intervention group will benefit from a minimum of 2 prone position sessions during the day. The patient should lie in prone position for a minimum of 2 sessions during the daytime, with an objective of a cumulative time of at least 2.5 hr in the prone position during the daytime. Patients are encouraged to lie in the prone position as frequently and for as long as feasible, as soon as possible after randomization. They are assisted during positioning and given every necessary accessory to be as comfortable as possible (pillows, cushions, foam wedges).

This study is design to be pragmatic without disturbing or increasing the management of medical wards in the present context. It will not be possible to monitor the duration of PP at night because of overloading caregivers already under pressure during the pandemic.

Clinical and demographic data in relation to the hospitalization will be collected. No major physiological measurements will be performed in relation to the prone position because of the pragmatic concern of saving the time of caregivers during this epidemic period.

## Research-Specific Procedures

### Prone position

1. Preparation

Check that all cables and lines (if present) are mobile enough to allow the patient to perform the self-prone position.

Explain the procedure to the patient:

- Perform a minimum of 2 prone position sessions during the daytime.
- With an objective of a cumulative time of at least 2.5 hr in the prone position during the daytime. The objective is to lie as much as possible in the prone position if it is possible.
- Patients will be able to move their head as they desire.
- The leg can be slightly bent to discharge the hip.
- Patients can change their bent leg as they wish while the spine remains free.
- The arms can be along the body or one arm can bent or both arms can be bent as in the picture below.


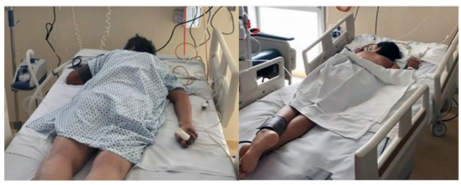
The position should be as comfortable as possible for the patient.

1. How to process the positioning

- Note the date and time of the beginning of the prone position in the notebook (by the patient or by a staff member).
- Ask the patient to lie on the stomach and place the barrier to prevent a potential fall out of bed.
- Make sure the patient is comfortable and explain that they can move themselves in the bed but the back should always be kept free to avoid compression of the lungs in the back region.
- Patients can turn slightly to a more comfortable position.
- The call bell is given to the patient so that they can call a staff member for assistance before each position change.
- Explain to the patient to not position themselves in the PP or supine position but use the call bell so that someone can help and set the barriers (the patient is informed that they can call at any time to move around or go to the toilet and that the barriers are an essential safety measure for the study; if the patient perfectly self-positions in the prone position, barriers would be removed after the investigator’s decision; this must be recorded and justified in the medical record).
- Note the date and time of the end of the prone position in the notebook (by the patient or by a staff member).

Each day of hospitalization, at least two PP sessions during the day, with the objective to spend as much time as possible in PP if tolerated and a minimum of 2.5 hr of cumulative time in PP during the day.

A session of PP as long as possible at night. Patients will be encouraged to sleep in PP as much as possible at night, knowing that monitoring the number of hours will not be feasible in COVID-19 medical wards in the current context.

Bedridden patients who are not able to self-position to the prone position or with minimal assistance of one person will not be included in the study.

Barriers will be positioned to avoid the potential fall out of bed.

The patient will be allowed to choose the best PP as long as the back of the thorax is not compressed by the bed.

### Semi-sitting in a bed or chair

The semi-sitting position in the bed is a minimum of 30° inclination, not more than 60% to 70% to avoid compression of the abdomen.

The patient can be installed in a chair if they wish. In this case, the position will be mentioned in the notebook.

The prone position is not authorized during the daytime. It is allowed at night only if it is the natural sleeping position.

Lateral position is authorized outside the intervention periods if the patient wishes and will be recorded.

### Oxygen therapy modalities:

All oxygen therapies will be allowed: nasal cannula, facemask (with moderate or high oxygen concentration) and high-flow oxygen therapy.

In the intervention group: the clinician is free to choose the best modality taking into account the needs of the patient, and the interface should be the same before and during the prone position session.

In both groups: clinicians will be free of the indication for oxygen therapy, the modalities for weaning the oxygen therapy or changing the interface. Generally, oxygen therapy should administered with SpO_2_ <92% on room air, and its flow rate should be maintained at the minimum level to obtain SpO2 ≥ 92% (High Council of Public Health/*Haut Conseil de la santé publique relatif aux recommandations thérapeutiques dans la prise en charge du COVID-19*, complémentaire à l’avis du 5 mars 2020, 23 mars 2020; disponible à l’adresse: https://www.hcsp.fr/Explore.cgi/AvisRapports).

### Blood samples

No blood samples are required for the trial.

## Trial progress

### Inclusion

The screening log will be established by the investigators of each center. From this list, patients who are eligible (meeting all the inclusion criteria and none of the exclusion criteria) will be identified as well as the included patients. The preferential sleeping position (prone, supine or lateral position) will be reported to determine any potential bias of selection. When a patient has all the inclusion criteria and none of the exclusion criteria, a member of the study will propose the study to the patient. For women of childbearing age, we will perform a beta-HCG test to exclude pregnancy.

If after information is given and a time for reflection of 2 hr, written consent is obtained, the patient will be included in the study. Randomization will be performed on a dedicated website after a standardize SpO_2_ measurement (5-L oxygen mask test with a face mask for 5 min). The investigator will note the date, time and group of inclusion in the patient’s record. The patient will be considered included once they have given consent.

NB: Patients may be included without an exclusion period (see paragraph 11.4) in other studies, whether comparative or not, with or without drugs, except for a study comparing prone position outside ICUs.

### Patient follow-up

In both groups, the rate of patients usually sleeping in the prone position will be collected at the beginning of the study. These data will be considered in the data analysis.

After randomization, the intervention will be performed according to the procedure detailed above.

The criteria for prone position intolerance are as follows:

- feeling of respiratory discomfort or any other uncomfortable feeling for the patient.

If any of the above criteria appear, we will record them in the specific case report form.

The standard surveillance will be applied by staff members of the enrolling wards. The staff will be free to consider whether a patient should return to the supine position and/or increase the surveillance (e.g., more frequent monitoring of blood pressure, respiratory rate or SpO_2_). It will be recorded in the case report form (CRF).

In the interventional group, an evaluation of SpO_2_ will be performed at the first session (to evaluate the clinical improvement after the first session):

- SpO_2_ measurement before PP
- SpO_2_ measurement just before the return to supine position at the end of the first session.

| *Actions* | *Day 0*  *(****Inclusion visit)*** | *Positions* | *End of treatment*  *(hospital discharge or intensive care unit with mechanical ventilation)* |
| --- | --- | --- | --- |
| *Diagnosis of COVID-19* | *X* |  |  |
| *Written consent of the patient* | *X* |  |  |
| *Fax of inclusion to the research department* | *X* |  |  |
| *Medical history* | *X* |  |  |
| *Usual sleeping position* | *X* |  |  |
| *Positioning techniques (PP/semi-sitting)* |  | *X* |  |
| *Improvement of 2 points on the WHO ordinal scale* |  |  | *X* |
| *Adverse events* | *X* | *X* | *X* |

## Research duration

Inclusion period: Epidemic COVID-19 period

Duration of intervention: the study intervention will stop when one of the events is reached: initiation of non-invasive or invasive ventilation, death, or patient still hospitalized at day 28 without event. Patients hospitalized for > 28 days will continue to be followed in the study (rate of use of resuscitation, rate of use of non-invasive or invasive ventilation, death).

The duration of participation for each patient corresponds to the hospitalization duration (all wards combined), subject to AEs that would require a longer follow-up.

Total duration of participation in the study for the patient: length of hospitalization

Total duration of the study: 24 months

## Measures to reduce and avoid bias

### Randomization

Randomization will be centralized via a website.

Randomization will be stratified according to the following three parameters:

- **According to the center**
- **Body mass index (BMI) < 30 kg.m^-2^ or not**
- **SpO_2_ (measured by pulse oximetry) < 95% or ≥ 95% on 5-L/min test for 5 min before randomization**

How SpO_2_ will be measured for stratification will be standardized: in patients already included but not yet randomized, SpO_2_ will be systematically measured after 5 min of 5-L/min nasal O_2_ therapy with a moderate concentration mask.

A randomization list will be established beforehand by a clinical study technician, by permuted blocks, whose size will remain unknown to the investigators. The strata are as follows:

**1. BMI < 30 kg.m^-2^ and SpO_2_ ≥ 95%**

**2. BMI < 30 kg.m^-2^ and SpO_2_ < 95%**

**3. BMI ≥ 30 kg.m^-2^ and SpO_2_ ≥ 95%**

**4. BMI ≥ 30 kg.m^-2^ and SpO_2_ < 95%**

The treatment arm will be assigned by randomization.

### Blinding methods

Blinding the patients and the investigator is not technically feasible.

## Data input in the CRF used as source data

| **Evaluations** | **Variables** |
| --- | --- |
| - Duration of prone position per session  - Number of prone position sessions  - Intolerance to prone position  If yes, specify the reason | Minutes  Continuous  Yes/No  In clear |
| - Semi-sitting in a chair  - Semi-sitting in bed  - Walking around  - Lateral position | Yes/No  Yes/No  Yes/No  Yes/no |
| - In interventional group: response to PP at first session  SpO_2_ before PP  SpO_2_ just before the return to the supine position | %  % |
| - 7-level ordinal scale (appendix 4) | 1 to 7 |

All other data will be collected from the patient’s hospital record.

## Rules for definitive or temporary termination

### Withdrawal of a patient’s participation in the research

Patients will be able to withdraw their consent and to leave the trial at any time and for any reason. In the case of premature withdrawal, the investigator must specify the reason.

The investigator will be able to definitively or temporarily stop the patient’s participation in the trial for any reason according to what is in the patient’s best interests, especially in the case of serious AEs.

The patient’s withdrawal shall not affect their standard of care for their pathology. In the case of an AE, whether serious or non-serious, specific follow-up can be considered, depending on the severity. The supervisory board shall then determine the conditions of follow-up on a case-by-case basis.

### Termination of part of the research or the research as a whole

The study can be suspended or prematurely interrupted in the case of unexpected serious AEs, requiring examination of the outcome for all patients already included.

If PP has led to the occurrence of cardiac arrest, we will report this to the sponsor who can suspend the study if they consider it necessary.

Likewise, unexpected events that would lead to the impossibility to achieve the primary and secondary endpoints may lead the sponsor to interrupt the study prematurely.

Orléans Regional Hospital reserves the right to interrupt the study at any time if the inclusion objectives are not reached.

# Drugs administered

## Authorized treatments

All

## Unauthorized treatments

None

## Emergency treatment

If PP induces a major decrease in SpO2 for more than 5 min or a non-resolving tachycardia despite a return to the classical sitting position or an adaptation of the oxygenation or non-invasive ventilation parameters by the clinician in charge of the patient, we will record this event as a non-serious AE if the supine position improves this parameter. However, if, despite the supine positioning, the patient does not show improvement of respiratory parameters, we will record a serious AE related to the PP.

If the respiratory signs deteriorate significantly, the use of non-invasive ventilation with 2 pressure levels or intubation will be possible if the clinician in charge of the patient considers it necessary. If intubation is considered unreasonable because of the evolution of the disease and vital parameters, although the patient would have initially received treatment in the ICU, appropriate care will be implemented for comfort in accordance with the practices of each department.

# Safety assessment

## Definitions

- **Adverse event (AE)**: any untoward occurrence in a patient or clinical investigation subject who is the subject of research involving humans. The event does not necessarily have a causal relationship with this treatment.
- **Adverse effect**: undesired harmful effect resulting from the intervention
- **Serious AE**: An adverse event or suspected adverse reaction is considered "serious" if, in the view of either the investigator or sponsor, the following outcomes occur:
  - Death
  - A life-threatening AE
  - A persistent or significant incapacity or substantial disruption of the ability to conduct normal life functions
  - Inpatient hospitalization
  - Prolongation of existing hospitalization
  - Congenital anomaly/birth defect
  - Requires intervention to prevent permanent impairment or damage
- **Unexpected AE**: adverse effect that is not consistent with the information given in the investigator’s information sheet
- **New information**: Any new information that may lead to a re-evaluation of the risk/benefit ratio of the research, change the research or stop or modify the research.

## Adverse event reporting

The monitoring and reporting of incidents and the risk of incidents resulting from participation in the trial will be declared via the Orléans Regional Hospital regular vigilance network with the following specificities.

##### Related to protocol:

The following event will automatically be considered as a serious AE of special interest:

- For prone position group:
  - Cardiac arrest
  - Persistent desaturation despite a return to the previous position without desaturation or adaptation of oxygenation
  - Unresolved hypotension by a return to the previous position without hypotension
  - Bradycardia < 45 bpm persisting for > 1 min
  - Catheter disconnection
  - Pressure point in the areas at risk
  - Adverse event exclusively due to the hospitalization
  - Catheter disconnection without clinical impact
  - Sinus tachycardia
  - Transitory desaturation resolving within 3 min
  - Death not related to prone position

Only cardiac arrest during a prone position session is subject to immediate reporting to the sponsor, using the form provided for this case by fax to 02 38 74 43 64 or by e-mail to [recherche.clinique@chr-orleans.fr](mailto:recherche.clinique@chr-orleans.fr).

The investigator must also attach the following to the cardiac arrest report whenever possible:

- A copy of the prolongation of hospitalization
- A copy of the autopsy report if applicable
- A copy of all results of additional exams including relevant negative results, with the normal laboratory values
- Any other document considered useful and relevant

These documents will be anonymized and will identified by the identification number of the patient.

ARDS induced by COVID-19 is a life-threatening disease; the risk of death is higher than in the standard population hospitalized in the enrolling departments. We will not consider the death as an AE unless it is attributable to one of the AEs listed above in association with the study’s intervention.

***How to deal with an AE:***

If an AE occurs, the investigator may:

- Continue the strategy
- Temporally or permanently stop the strategy

In addition, monitoring will be intensified and all measures to prevent the occurrence of an AE will be taken by the investigator with an immediate and explicit notification to the sponsor.

### Conditions and duration of follow-up for an AE

Each adverse event will be followed up until its complete resolution (stabilization to a level considered acceptable by the investigator, or a return to the previous state), even if the patient has left the trial.

### Supervisory board

No supervisory board was considered necessary.

# Statistics

Statistical analysis will be performed by Dr Thierry Boulain, using R v.3.6.0 *(R Foundation for Statistical Computing).*

## Statistical methods

A detailed analysis plan will be developed prior to the start of the study. The plan will be modified, if necessary, before the statistician is unblinded.

Oatient characteristics will be described by randomization group as number and percentages for categorical variables and mean (standard deviation) or median (25th and 75th percentile) for continuous variables, depending on their distribution.

The main analysis will be conducted on an intention-to-treat basis. However, data for patients withdrawing their consent during the study, in accordance with French law, will not be analyzed.

The primary endpoint (percentage of patients requiring intubation or non-invasive ventilation at 2 pressure levels, or who died within 28 days of inclusion) will be compared between the 2 groups (PP and usual care) by a Mantel-Haenszel 2-test stratified on the stratification variables #2 and #3 (SpO2 and BMI). The effect of the intervention will be expressed in terms of absolute risk difference and its 95% confidence interval (CI). The result of this analysis will be confirmed by a mixed-effect logistic regression analysis, with the recruiting center as the random effect and the study intervention and the stratification variables 2 and 3 as fixed effect variables. Interaction terms between the stratification variables and the intervention will be introduced into the model and retained only if they show a significant effect on the frequency of the outcome. Logistic regression analysis will be used to estimate the odds ratio and its 95% CI.

The binary secondary endpoints (intubation rate, rate of non-invasive ventilation at 2 pressure levels, rate of transfer to ICU, mortality) will be compared between the groups using the same method.

The time to clinical improvement (decrease of 2 points on the WHO scale) will be compared between groups by log rank test and presented as Kaplan-Meier curves. The difference in risk between the groups will be expressed as a hazard ratio (HR) and its 95% CI. This analysis will be secondarily adjusted for stratification variables, and possibly other covariables that would be misbalanced between groups, using a Cox proportional model provided the proportional assumption is verified.

The durations (of oxygen therapy, non-invasive ventilation, hospitalization), will be compared between groups by Mann-Whitney U test adjusted for stratification variables [KAWAGUCHI; Statistics in Biopharmaceutical Research; 2011]. The difference in duration (of oxygen therapy, non-invasive ventilation, hospitalization) between groups will be expressed by its median and 95% CI obtained by bootstrapping (2000 unstratified samples).

We will conduct a per-protocol analysis including only patients of the intervention group who laid in a prone position for at least 2 hr each day, and patients of the usual care group who never laid in a prone position.

The above analyses will be repeated in prespecified subgroups formed according to the stratification variables #2 and #3 (SpO2 and BMI).

No interim analysis is planned.

## Number of participants to be included in the research

With a rate of lost to follow-up of < 2%, a population of 268 patients would guarantee a power of at least 80% to demonstrate at the 2-sided alpha risk of 5%, a difference of 10% to 14% between the 2 groups concerning the composite primary endpoint (4% vs 14% or 10% vs 24%). The number of patients to be recruited is fixed at 268.

## Considered degree of statistical significance

P<0.05

## Statistical research termination criterion

None.

## Management of missing, unused or invalid data

In this trial and considering that the protocol will be respected, no missing data are expected for the intervention, stratification and endpoint variables. Vital status at 28 days for patients discharged before D28 will be collected by telephone call by a member of the research team (as a precaution, the town of birth of each included patient will be noted at inclusion in order to collect vital status from city halls if necessary). Patients who withdraw their consent will be excluded from the analysis as required by French law, and it is not planned to replace them by imputation for the main analysis. However, the number of patients withdrawing their consent will be analyzed to assess whether it does not reveal an imbalance between the 2 randomization groups, which could be related to a less tolerance of either arm.

## Selection of participants to be included in the analysis

The main analysis will be conducted on an intention-to-treat basis. However, data for patients withdrawing their consent during the study, in accordance with French law, will not be analyzed.

A per-protocol analysis including only patients who performed at least 2 hr of PP during the day in the PP group and patients in the usual care group who did not perform PP, is planned.

# Data and source document access rights

## Data access rights

In accordance with Good Clinical Practice (GCP):

- The sponsor is in charge of obtaining the approval of all parties involved so that direct access is ensured to the sites where the trial takes place, to the source data, to the source documents and to the reports. The purpose of this being that the sponsor will be able to carry out quality control and audit.

- Investigators shall provide the persons in charge of follow-up, quality control or audits with the research documents and individual data strictly necessary for those purposes, according to the provisions of French Law (articles L.1121-3 and R.5121-13 of the French Public Health Code).

## Source documents

Source documents are defined as any documents or original objects that could be used to prove the existence or accuracy of data or any events occurring during the study. They will be kept for 25 years by the investigator or the hospital if it is a medical hospital record.

All the information requested for the protocol will be recorded in the CRF in paper format.

## Data confidentiality

In accordance with the provisions concerning the confidentiality of the data made available to the persons in charge of quality control of biomedical research (article L.1121-3 of the French Public Health Code), in accordance with the provisions concerning the confidentiality of the information on the nature of the products, trials, the consenting parties and the results (article R. 5121-13 of the French Public Health Code), persons with direct access shall take the necessary precautions to ensure the confidentiality of the information of the products, trials, the consenting parties, their identity and the results.

Those persons, and also the investigators themselves, are bound by professional secrecy ( articles 226-13 and 226-14 of the French penal code).

During the biomedical research or at its conclusion, the data collected on the consenting parties, sent by the investigators to the sponsor (or any other specialized operator) shall be anonymized.

Investigators should not, under any circumstances, reveal the name or contact details of the individuals concerned.

Only the first letter of the last name and the first letter of the first name of the patient will be registered, along with a coded number specific to the trial indicating the order of inclusion.

The sponsor shall ensure that each consenting party has given their written approval for access to their own individual data, strictly necessary for the research quality control.

# Quality control and assurance

A clinical research associate (CRA) assigned by the sponsor will ensure the proper conduct of the study, collection of written data, their documentation, recording and reporting, in accordance with the standard operating procedures implemented in the Orleans Regional Hospital, in accordance with GCP and with existing legislative provisions.

The investigator and their team members agree to make themselves available for the regular quality control visits carried out by the CRA. During these visits, the following elements will be revised:

- Written consent signed by the patient
- Adherence to the study protocol and the procedures defined
- Quality of the collection of the data in the CRF: accuracy, missing data, consistency with the source documents

Furthermore, the investigators undertake to agree to quality assurance audits conducted by the sponsor and to inspections carried out by the competent authority. All the data, documents and reports may be subject to audits and regulatory inspections without the possibility of opposing medical secrecy

# Ethical considerations

## Independent Ethics Committee

The trial protocol and information leaflet will be submitted to an independent ethics committee selected by drawing lots, for its opinion.

The notification of the favorable opinion from the independent ethics committee will be sent to the sponsor and to the competent authority.

For the Monaco center, the study documents will also be submitted to the Consultative Committee on Ethics in Biomedical Research (CCEMRB), which is the local authority of the Principality of Monaco. The study can only begin after their approval.

## Substantial amendments

In case of substantial modification to the protocol by the investigator, this modification will be approved by the sponsor. The sponsor will need to obtain a preliminary favorable opinion from the independent ethics committee before the substantial amendment can be made. A new consent will be collected from the participants if necessary.

## Patient information and consent form

Patients will receive comprehensive and accurate information about the study objectives, their right to refuse to participate in the trial or their possibility to withdraw from the study, in terms they are able to understand. All of this information will feature in the information leaflet given to the patient. The patients consenting to participation will have to date and sign the consent form. Refusal or consent will be specified by the investigator in the patient’s medical record.

All of this information will feature in the information leaflet given to the patient. The written consent of the patient will be obtained by the investigator, in accordance with the Clinical trial involving human participants N°2 law, prior to final inclusion in the study.

The information letter and consent for patients in France are already validated by the CPP. The letter of information and consent for patients in the Principality of Monaco will be examined by the CCEMRB (compliance with the Monegasque law).

A consent form will be completed by the investigator and by the patient in order to trace the agreement of the patient's consent. A copy of the information and consent form will be given to the patient, the investigator will keep the original.

## Definition of the exclusion period

Patients included in the study will be allowed to participate in another study EXCEPT a study assessing prone position in the COVID-19 in medical wards (i.e., outside the ICU) during the 28-day follow-up. If the patient is transferred to an ICU, the study intervention will stop and the ICU practitioners will be free to care for the patient as they wish. During the SARS-CoV-2 epidemic, in the absence of extensive knowledge about the best management modalities, clinical research evaluating the full range of drug and non-drug strategies is an international priority.

## Compensation

NA

## Registration of persons consenting to biomedical research on the national data file

NA

# Data and document use and storage

## Case report form (CRF)

An electronic CRF (eCRF) will be developed. However, a notebook will be provided with the data to be collected in real time.

All information required by the protocol must be recorded in the notebook and an explanation must be provided for each missing data. Data should be collected as they are obtained and transcribed into these notebooks in a neat and legible manner.

Erroneous data found on the CRFs should be clearly crossed out and the new data copied next to the crossed-out information, with initials, date, and possibly a justification by the investigator or authorized person who made the correction.

In the context of the COVID-19 epidemic, all paper documents will be placed in a plastic bag at the patient's discharge and kept for 5 days before opening it for analysis and data entry.

## Data input and data production

Data will be entered into an eCRF medsharing by the investigation team of each departments involved in the study.

## French data protection commission (CNIL-France)

This study is related to the "Reference Methodology" (MR-001) in application to Deliberation No. 2018-153 of May 3, 2018 approving a reference methodology for the processing of personal data implemented in the context of research in the health field with the collection of consent from the person concerned and repealing Deliberation No. 2016-262 of July 21, 2016.

Orleans Regional Hospital, the study sponsor, signed an undertaking to comply with this "Reference Methodology" on March 10, 2017.

Considering the automated data recording on Monegasque territory (eCRF), the file will be submitted to the Commission de Contrôle des Informations Nominatives (CCIN) of Monaco for advice.

## Archiving

The following documents will be archived under the study title in the Research Department of Orleans Regional Hospital until the end of the period of use.
The documents include:
→ Protocol, appendices and potential amendments
→ Information leaflet
→ Individual data (authenticated copies of raw data)
→ Follow-up documents
→ Statistical analysis
→ Final study report
At the end of the practical use period, all archiving documents will be placed under the responsibility of the sponsor for 25 years after the end of the study, in accordance with institutional practices.

Documents shall not be moved or destroyed without the sponsor’s agreement. At the end of the 25-year period, the sponsor shall be contacted concerning destruction. All data, documents and reports will be subject to audit or inspection.

# Funding and insurance

## Budget

Costs related to this research will be covered by the sponsor and include the following:

• Specific insurance

• Data management: eCRFs, data management, statistical analysis

• Time spent for data input

• Variable costs: stationary

## Insurance

The Orleans Regional Hospital has taken out insurance with SHAM for the duration of the study to cover its own civil liability and that of any physicians involved in the trial. It will also insure full compensation for any damages related to the research caused to persons participating in the study and their beneficiaries, unless it can otherwise be proven that it is not liable for the said damages, unless proven against the physician that the damage is not attributable to their fault or that of any intervener, without being able to oppose the fact of a third party or the voluntary withdrawal of the person who had initially consented to participate in the research.

# Practical aspects of the study

The epidemic of COVID-19 has led to a massive influx of patients in hospitals. To date, for the Orléans Regional Hospital, we do not have precise figures for patients meeting the inclusion criteria, but the multicenter aspect will allow us to quickly include the number of patients necessary for statistical analysis.

Patient mobilization is routine in the participating hospital departments. All the centers have medical beds adapted (barriers/tilt of the backrest etc.) to the practice of PP in spontaneously breathing patients.

The medical departments participating in this project have been conducting and participating in research projects for years and the physicians are trained in GCP for research.

For the initiation of the centers and the verification of the data, a CRA is available within the research department of the Orléans Regional Hospital Centre.

Therefore, we have all the operational skills to carry out this study in optimal conditions of safety, ethics and know-how.

# Rules relating to publication

The 2 main authors will be Guillaume Fossat and Mai-Anh Nay. The penultimate author will be Thierry Boulain. The rest of the authorship will depend on the number of inclusions in each center. Communications and scientific reports about the study will be issued under the responsibility of the main investigator and project coordinator with the agreement of the other investigators in charge. The co-authors of the report and publications will be the investigators and clinicians involved, to an extent proportionate to their contribution to the study, and also the associated biostatistician and research scientists.

Publication rules will follow the international guidelines (N Engl J Med, 1997; 336: 309-315).

The trial will be registered on a free access website (ClinicalTrials.gov) before the inclusion of the first patient in the study.

# Sources

1. Murthy S, Gomersall CD, Fowler RA. Care for Critically Ill Patients With COVID-19. JAMA. Published online March 11, 2020. doi:10.1001/jama.2020.3633
2. Huang C, Wang Y, Li X, Ren L, Zhao J, Hu Y, et al. Clinical features of patients infected with 2019 novel coronavirus in Wuhan, China. Lancet. 15 févr 2020;395(10223):497‑506.
3. Zhou F, Yu T, Du R, Fan G, Liu Y, Liu Z, et al. Clinical course and risk factors for mortality of adult inpatients with COVID-19 in Wuhan, China: a retrospective cohort study. Lancet. 28 mars 2020;395(10229):1054‑62.
4. Wu C, Chen X, Cai Y, Xia J, Zhou X, Xu S, et al. Risk Factors Associated With Acute Respiratory Distress Syndrome and Death in Patients With Coronavirus Disease 2019 Pneumonia in Wuhan, China. JAMA Intern Med. 13 mars 2020;
5. Guan W, Ni Z, Hu Y, Liang W, Ou C, He J, et al. Clinical Characteristics of Coronavirus Disease 2019 in China. N Engl J Med 28 févr 2020
6. Guo T, Fan Y, Chen M, et al. Cardiovascular Implications of Fatal Outcomes of Patients With Coronavirus Disease 2019 (COVID-19). JAMA Cardiol.
7. Wang D, Hu B, Hu C, Zhu F, Liu X, Zhang J, et al. Clinical Characteristics of 138 Hospitalized Patients With 2019 Novel Coronavirus-Infected Pneumonia in Wuhan, China. JAMA. 7 févr 2020;
8. Arentz M, Yim E, Klaff L, Lokhandwala S, Riedo FX, Chong M, et al. Characteristics and Outcomes of 21 Critically Ill Patients With COVID-19 in Washington State. JAMA. 19 mars 2020;
9. Grasselli G, Pesenti A, Cecconi M. Critical Care Utilization for the COVID-19 Outbreak in Lombardy, Italy: Early Experience and Forecast During an Emergency Response. JAMA. 13 mars 2020;
10. The ARDS Definition Task Force*. Acute respiratory distress syndrome: The berlin definition. JAMA. 20 juin 2012;307(23):2526‑33.
11. Aliaga M, Forel J-M, De Bourmont S, Jung B, Thomas G, Mahul M, et al. Diagnostic yield and safety of CT scans in ICU. Intensive Care Med. mars 2015;41(3):436‑43.
12. Umbrello M, Formenti P, Bolgiaghi L, Chiumello D. Current Concepts of ARDS: A Narrative Review. Int J Mol Sci. 29 déc 2016;18(12):64.
13. Guérin C, Reignier J, Richard J-C, Beuret P, Gacouin A, Boulain T, et al. Prone Positioning in Severe Acute Respiratory Distress Syndrome. N Engl J Med. 6 juin 2013;368(23):2159‑68.
14. Hoste EAJ, Roosens CDVK, Bracke S, Decruyenaere JMA, Benoit DD, Vandewoude KHDK, et al. Acute Effects of Upright Position on Gas Exchange in Patients With Acute Respiratory Distress Syndrome. J Intensive Care Med. janv 2005;20(1):43‑9
15. Richard J-CM, Maggiore SM, Mancebo J, Lemaire F, Jonson B, Brochard L. Effects of vertical positioning on gas exchange and lung volumes in acute respiratory distress syndrome. Intensive Care Med. 29 sept 2006;32(10):1623‑6
16. Robak O, Schellongowski P, Bojic A, Laczika K, Locker GJ, Staudinger T. Short-term effects of combining upright and prone positions in patients with ARDS: a prospective randomized study. Crit Care. 2011;15(5):R230.
17. Dellamonica J, Lerolle N, Sargentini C, Hubert S, Beduneau G, Marco FD, et al. Effect of different seated positions on lung volume and oxygenation in acute respiratory distress syndrome. Intensive Care Med. juin 2013;39(6):1121‑7.
18. Ding L, Wang L, Ma W, He H. Efficacy and safety of early prone positioning combined with HFNC or NIV in moderate to severe ARDS: a multi-center prospective cohort study. Crit Care. 30 2020;24(1):28.
19. Gattinoni L, Taccone P, Carlesso E, Marini JJ. Prone position in acute respiratory distress syndrome. Rationale, indications, and limits. Am J Respir Crit Care Med. 1 déc 2013;188(11):1286‑93.
20. Pérez-Nieto, O.R., Guerrero-Gutiérrez, M.A., Deloya-Tomas, E. *et al.* Prone positioning combined with high-flow nasal cannula in severe non-infectious ARDS. *Crit Care* **24,**114 (2020). <https://doi.org/10.1186/s13054-020-2821-y>
21. Zhou S, Wang Y, Zhu T, Xia L. CT Features of Coronavirus Disease 2019 (COVID-19) Pneumonia in 62 Patients in Wuhan, China. AJR Am J Roentgenol. 5 mars 2020;1‑8.
22. Zhou P, Yang XL, Wang XG, Hu B, Zhang L, Zhang W, Si HR, Zhu Y, Li B, Huang CL, Chen HD, Chen J, Luo Y, Guo H, Jiang RD, Liu MQ, Chen Y, Shen XR, Wang X, Zheng XS, Zhao K, Chen QJ, Deng F, Liu LL, Yan B, Zhan FX, Wang YY, Xiao GF, Shi ZL. A pneumonia outbreak associated with a new coronavirus of probable bat origin. Nature. 2020 Mar;579(7798):270-273.
23. Jia HP, Look DC, Shi L, Hickey M, Pewe L, Netland J, Farzan M, Wohlford-Lenane C, Perlman S, McCray PB Jr. ACE2 receptor exprèsion and severe acute respiratory syndrome coronavirus infection depend on differentiation of human airway epithelia. J Virol. 2005 Dec;79(23):14614-21.
24. Khan A, Benthin C, Zeno B, Albertson TE, Boyd J, Christie JD, Hall R, Poirier G, Ronco JJ, Tidswell M, Hardes K, Powley WM, Wright TJ, Siederer SK, Fairman DA, Lipson DA, Bayliffe AI, Lazaar AL. A pilot clinical trial of recombinant human angiotensin-converting enzyme 2 in acute respiratory distress syndrome. Crit Care. 2017 Sep 7;21(1):234.
25. Juillerat-Jeanneret L. The Other Angiotensin II Receptor: AT(2)R as a Therapeutic Target. J Med Chem. 2020 Mar 12;63(5):1978-1995.
26. Li Y, Zeng Z, Li Y, Huang W, Zhou M, Zhang X, Jiang W. Angiotensin-converting enzyme inhibition attenuates lipopolysaccharide-induced lung injury by regulating the balance between angiotensin-converting enzyme and angiotensin converting enzyme 2 and inhibiting mitogen-activated protein kinase activation. Shock. 2015 Apr;43(4):395-404.
27. Khan A, Benthin C, Zeno B, Albertson TE, Boyd J, Christie JD, Hall R, Poirier G, Ronco JJ, Tidswell M, Hardes K, Powley WM, Wright TJ, Siederer SK, Fairman DA, Lipson DA, Bayliffe AI, Lazaar AL. A pilot clinical trial of recombinant human angiotensin-converting enzyme 2 in acute respiratory distress syndrome. Crit Care. 2017 Sep 7;21(1):234.
28. Vaduganathan M, Vardeny O, Michel T, McMurray JJV, Pfeffer MA, Solomon SD. Renin–Angiotensin–Aldosterone System Inhibitors in Patients with Covid-19. N Engl J Med [Internet]. 30 mars 2020 [cité 1 avr 2020]; Disponible sur: <https://doi.org/10.1056/NEJMsr2005760>
29. Kawaguchi A, Koch GG, Wang X. Stratified Multivariate Mann-Whitney Estimators for the Comparison of Two Treatments with Randomization Based Covariance Adjustment. *Statistics in Biopharmaceutical Research*. 2011 ;3(2):217-231.
30. Cao B, Wang Y, Wen D, Liu W, Wang J, Fan G, et al. A Trial of Lopinavir–Ritonavir in Adults Hospitalized with Severe Covid-19. New England Journal of Medicine. 18 mars 2020;0(0)

# Appendices

Appendix 1: Illustration of prone positioning (PP) in spontaneously breathing patient

Appendix 2: PP schedule collection

Appendix 3: Example of a follow-up worksheet in the control group

Appendix 4: WHO ordinal scale (according to CAO ; N Eng J Med ; 2020)

APPENDIX 1: Illustration of prone positioning (PP) in spontaneously breathing patient

1. **PUT THE BARRIERS**
2. **KEEP THE CATHETERS AND LINES FREE AROUND THE PATIENT**
3. **ASK THE PATIENT TO LIE ON PRONE POSITION**
4. **NOTE THE START OF PRONE POSITION**
5. **GIVE PATIENT THE CALLBELL for call the staff if necessary**
6. **EXPLAIN TO THE PATIENT THAT THEY MUST LIE 30 min to 1 HOUR PER SESSION (minimum)**


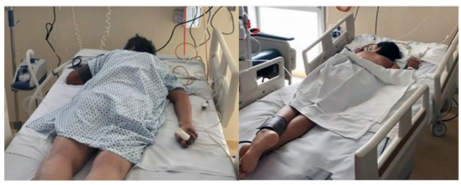


APPENDIX 2: PP schedule collection

**Each mobilization (time and duration) during daytime is recorded in a notebook by the patient or by a staff member**

**If you feel tired or your breathing becomes more difficult during your prone position, you can go back to supine. Once you are repositioned, PLEASE NOTE THE TIME OF YOUR RETURN TO SUPINE POSITION.**

| **PP Session n°1** |  |  |  |
| --- | --- | --- | --- |
| Date: | Start time: | End time: | Intolerance: Yes/No  Cause (in plain): |
| **PP Session n°…** |  |  |  |
| Date: | Start time: | End time: | Intolerance: Yes/No  Cause (in plain): |
| **PP Session n°...** |  |  |  |
| Date: | Start time: | End time: | Intolerance: Yes/No  Cause (in plain): |
| **PP Session n°…** |  |  |  |
| Date: | Start time: | End time: | Intolerance: Yes/No  Cause (in plain): |
| **PP Session n°…** |  |  |  |
| Date: | Start time: | End time: | Intolerance: Yes/No  Cause (in plain): |
| **PP Session n°…** |  |  |  |
| Date: | Start time: | End time: | Intolerance: Yes/No  Cause (in plain): |
| **PP Session n°…** |  |  |  |
| Date: | Start time: | End time: | Intolerance: Yes/No  Cause (in plain): |

APPENDIX 3: Example of a follow-up worksheet in the control group

| **Day 1: Date =** | | | |
| --- | --- | --- | --- |
| Chair  Yes/No | Semi-sitting position  Yes/No | Walking around  Yes/No (toilette, transfer alone) | Lateral position: Yes/No |
| **Day 2: Date =** | | | |
| Chair  Yes/No | Semi-sitting position  Yes/No | Walking around  Yes/No (toilette, transfer alone) | Lateral position: Yes/No |
| **Day 3: Date =** | | | |
| Chair  Yes/No | Semi-sitting position  Yes/No | Walking around  Yes/No (toilette, transfer alone) | Lateral position: Yes/No |
| **Day 4: Date =** | | | |
| Chair  Yes/No | Semi-sitting position  Yes/No | Walking around  Yes/No (toilette, transfer alone) | Lateral position: Yes/No |
| **Day 5: Date =** | | | |
| Chair  Yes/No | Semi-sitting position  Yes/No | Walking around  Yes/No (toilette, transfer alone) | Lateral position: Yes/No |
| **Day6: Date =** | | | |
| Chair  Yes/No | Semi-sitting position  Yes/No | Walking around  Yes/No (toilette, transfer alone) | Lateral position: Yes/No |

APPENDIX 4: WHO ordinal scale (according to CAO; N Eng J Med; 2020)

1. Not hospitalized, no limitations of activities
2. Not hospitalized, limitation on activities
3. Hospitalized, not requiring supplemental oxygen
4. Hospitalized, requiring supplemental oxygen
5. Hospitalized, on non-invasive ventilation or high flow oxygen devices
6. Hospitalized, on invasive mechanical ventilation or ECMO or both
7. Death
