# Supplementary material for: Prone position versus usual care in hypoxemic COVID-19 patients in medical wards: a randomised controlled trial
Source: Crit Care. 2023 Jun 17;27:240. doi: 10.1186/s13054-023-04529-z (PMC10276908; doi:10.1186/s13054-023-04529-z)
Supplement: Supplementary file 2 — Additional file 2. Supplementary files. [file 13054_2023_4529_MOESM2_ESM.docx]

Prone-position versus usual care in hypoxemic COVID-19 patients in medical wards: a randomized controlled trial.

**Authors**:

Mai-Anh Nay, Raphaël HINDRE, Christophe PERRIN, Jérémy CLÉMENT, Laurent PLANTIER, Aymeric SÈVE, Sylvie DRUELLE, Marine MORRIER, Jean-Baptiste LAINÉ, Léa COLOMBAIN, Grégory CORVAISIER, Nicolas BIZIEN, Xavier POUGET-ABADIE, Adrien BIGOT, Simon JAMARD, Elsa NYAMANKOLLY, Benjamin Planquette, Guillaume FOSSAT, Thierry BOULAIN.

**SUPPLEMENT 2**

- Investigators of the PROVID-19 trial, page 2
- Modification of the statistical plan, **eTable 1** and **eFigure 1**, page 3
- Improvement in the clinical WHO scale, **eFigure 2 and eTable 2**, page 6 and 7
- Per-protocol analysis, **eTable 3**, page 8
- Time-to-event exploratory analyses, **eFigures 3 to 13**, pages 9 to 17
- References, page 18

**INVESTIGATORS FROM EACH PARTICIPATING CENTRE:**

| **Centre** | **Investigator** |
| --- | --- |
| Department of Pneumology, CHR d’Orléans, Orléans, France | Sylvie DRUELLE, MD; Hugues MOREL, MD; Sophie MIRAN, SN |
| Department of Infectious and Tropical Disease, CHR d’Orléans, Orléans, France | Camélia GUBAVU, MD; Vincent RZEPECKI, MD; Aymeric SEVE, MD; |
| Department of Pneumology and Respiratory Functional testing, CHRU de Tours, Tours, France | Laurent PLANTIER, MD, PhD; Thomas FLAMENT, MD; Pascal MAGRO, MD; Julie MANKIKIAN, MD |
| Department of Internal Medicine, CHRU de Tours, Tours, France | Adrien BIGOT, MD; Maeva DIEU, SN; Nicole FERREIRA-MALDENT, MD; François MAILLOT, MD, PhD |
| Department of Infectious diseases, CHRU de Tours, Tours, France | Louis BERNARD, MD, PhD; François COUSTILLERES, MD; Simon JAMARD, MD; Adrien LEMAIGNEN, MD, PhD |
| Department of Internal Medicine and Infectious Disease, Hospital Dax Côte d’Argent, Dax, France | Elsa NYAMANKOLLY, MD; Katell ANDRE, MD; Maider LAUDA MAILLEN, MD; Anne-Hélène BOIVIN, PhD |
| Department of Internal Medicine and Infectious Diseases, Groupement Hospitalier La Rochelle Ré Aunis, La Rochelle, France | Xavier POUGET-ABADIE, MD |
| Department of Infectious Diseases, Centre Hospitalier Departemental de la Vendée, La Roche sur Yon, France | Orane COLIN, MD; Thomas, GEORGES, MD; Dominique, MERRIEN, MD; Marine MORRIER, MD; |
| Department of Infection and tropical diseases and pneumology, Perpignan Hospital Centre, Perpignan, France | Léa COLOMBAIN, MD; Cécile GRANGER, MD; Kevin BERTRAND, MD; Alexis REDOR, MD |
| Department of Pneumology, Centre hospitalier intercommunal de Cornouaille, Quimper, France | Nicolas BIZIEN, MD; Marie COIFFEY, MD |
| Department of respiratory medicine, Assistance publique des Hôpitaux de Paris AP-HP, Paris, France | Manil BENLOUNES, MD; Sixtine, DECAUT, MD; Hamer HAMDAN, MD; Raphael HINDRE, MD; Jean PASTRE, MD; Benjamin PLANQUETTE, MD, PhD; Vincent, ROTHSTEIN, MD; Solene VALERY, MD |
| Department of Internal Medicine and General Medicine, Centre Hospitalier Simon Veil, Blois, France | Jérémy CLEMENT, MD; Bertrand LIOGER, MD |
| Department of Infectious Diseases, Le Mans Hospital, Le Mans, France | Florence, DANGEUL, SN; François GOUPIL, MD; Jean-Baptiste LAINE, MD; Lucia PEREZ, MD |
| Department of Pneumology and Pneumo-Covid Unit, Centre Hospitalier Princesse Grace, Monaco, Monaco | Ryah FARHAD, MD; Cécile MAINCENT, MD; Christophe PERRIN, MD, PhD; Julien RENVOISE, MD |
| Department of pneumology, Centre hospitalier Bretagne Atlantique Vannes, France | Grégory CORVAISIER, MD; Audrey CREAC’H CADEC, MD; Thyphaine GUY, MD; |

**MODIFICATION OF THE STATISTICAL PLAN**

In the published statistical plan,^1^ it was planned to test the difference in the rate of the primary outcome occurrence between the groups of randomisation by using a Mantel-Haenzel chi-squared test stratified on stratification variables (initial SpO_2_ < or ≥95% and body mass index < or ≥30 kg/m^2^). However, the matrix of strata, which was a high-order contingency table, showed that 1) several medical wards did not recruit patients in one or two strata (see **eTable 1** below), 2) several medical wards did not randomise any patient in the prone positioning group or in the usual care group for one or several strata, 3) there were many empty cells in the contingency table, and 4) 4 medical wards (among 15) had a rate of treatment failure (primary outcome) of 0% (see e**Figure 1** below).

With the data at hand, we considered that testing the between-group difference while adjusting for variables of stratification could not rely on a Mantel-Haenzel chi-squared test.

Therefore, we opted for the use of a mixed-effect logistic regression (initially planned as a sensitivity analysis) to analyse the primary outcome. We entered the recruiting medical ward as a variable with a random effect and the group of randomisation and the stratification variables (SpO_2_ and body mass index) as variables with a fixed effect.

**eTable 1**: Counts of patients in subgroups according to centre, variables of stratification, group of randomisation and primary outcome

|  | Strata | | | | | | | | | | | | | | | |
| --- | --- | --- | --- | --- | --- | --- | --- | --- | --- | --- | --- | --- | --- | --- | --- | --- |
|  | BMI ≥30 kg/m^2^ & SpO2 ≥95% | | | | BMI ≥30 kg/m^2^ & SpO2 <95% | | | | BMI <30 kg/m^2^ & SpO2 ≥95% | | | | BMI <30 kg/m^2^ & SpO2 <95% | | | |
|  | **Prone position** | | **Usual care** | | **Prone position** | | **Usual care** | | **Prone position** | | **Usual care** | | **Prone position** | | **Usual care** | |
|  | **Primary outcome** | | **Primary outcome** | | **Primary outcome** | | **Primary outcome** | | **Primary outcome** | | **Primary outcome** | | **Primary outcome** | | **Primary outcome** | |
| Hospital departments | **no** | **Yes** | **no** | **Yes** | **no** | **Yes** | **no** | **Yes** | **no** | **Yes** | **no** | **Yes** | **no** | **Yes** | **no** | **Yes** |
| #1 | 2 | 0 | 2 | 0 | 0 | 1 | 1 | 0 | 4 | 0 | 4 | 0 | 3 | 0 | 3 | 0 |
| #2 | 4 | 1 | 2 | 2 | 0 | 0 | 0 | 0 | 6 | 1 | 6 | 0 | 0 | 0 | 0 | 0 |
| #3 | 3 | 0 | 1 | 1 | 0 | 0 | 2 | 0 | 6 | 0 | 5 | 1 | 1 | 0 | 3 | 0 |
| #4 | 1 | 0 | 0 | 0 | 0 | 0 | 0 | 0 | 1 | 0 | 1 | 0 | 1 | 0 | 1 | 0 |
| #5 | 0 | 1 | 1 | 0 | 2 | 0 | 0 | 0 | 1 | 0 | 0 | 0 | 1 | 0 | 0 | 0 |
| #6 | 0 | 0 | 0 | 0 | 0 | 0 | 0 | 0 | 1 | 0 | 1 | 0 | 0 | 1 | 0 | 0 |
| #7 | 0 | 0 | 0 | 0 | 1 | 0 | 1 | 0 | 2 | 0 | 1 | 1 | 1 | 0 | 0 | 0 |
| #11 | 2 | 0 | 1 | 0 | 0 | 0 | 0 | 0 | 4 | 1 | 6 | 0 | 0 | 0 | 0 | 1 |
| #12 | 1 | 0 | 0 | 0 | 3 | 0 | 2 | 0 | 0 | 0 | 2 | 0 | 1 | 0 | 0 | 0 |
| #14 | 1 | 0 | 2 | 0 | 0 | 0 | 0 | 0 | 3 | 0 | 0 | 0 | 0 | 1 | 0 | 0 |
| #15 | 4 | 0 | 3 | 1 | 1 | 2 | 3 | 1 | 10 | 0 | 8 | 3 | 8 | 8 | 13 | 4 |
| #17 | 4 | 0 | 3 | 0 | 1 | 1 | 1 | 1 | 4 | 0 | 4 | 0 | 2 | 0 | 2 | 0 |
| #18 | 2 | 0 | 2 | 1 | 0 | 0 | 0 | 0 | 4 | 1 | 4 | 0 | 0 | 0 | 0 | 0 |
| #19 | 3 | 0 | 4 | 0 | 0 | 0 | 1 | 0 | 11 | 0 | 11 | 0 | 2 | 0 | 3 | 0 |
| #20 | 0 | 0 | 2 | 0 | 1 | 0 | 0 | 0 | 3 | 0 | 3 | 0 | 0 | 0 | 0 | 0 |

Pink cells show that zero patients were recruited in 1 or 2 strata in several departments.

Yellow cells show that several departments did not randomise any patient in the prone position group or in the usual care group for one or several strata.

**eFigure 1: Rate of primary outcome by centre**

Black squares represent the percentage of treatment failure observed in each department. Bars surrounding the squares represent the Wilson 95% confidence interval

**RESULTS CONCERNING THE IMPROVEMENT ON THE CLINICAL WHO SCALE** ^2^

**eFigure 2: Cumulative incidence of 2-point improvement on the clinical WHO scale**


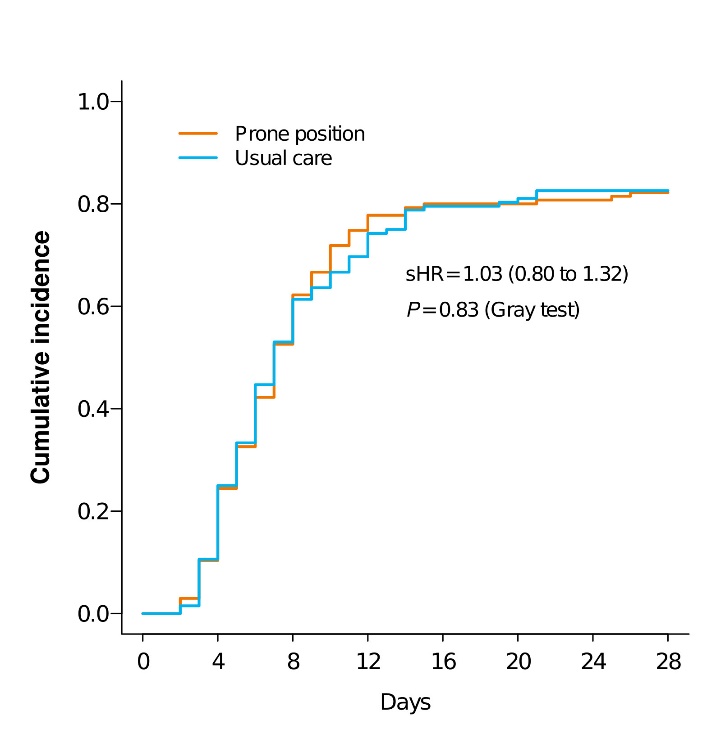


sHR, Subdistribution hazard ratio.

Two-point improvement on the clinical WHO scale was defined as a continuous uneventful improvement (i.e., without the need for escalating respiratory support [including increase of oxygen flow], from the date of inclusion to a 2-point decrease. The analysis used a competing risk approach (Fine and Gray model) with escalation of respiratory support as a competing event (and censoring if neither the 2-point improvement and escalation of respiratory support occurred before day 28), adjusted for body mass index and initial SpO_2_***.***

**eTable 2: Factors associated with the cumulative incidence of 2-point improvement on the clinical WHO scale**

|  | sHR (95%CI) | P-value |
| --- | --- | --- |
| Prone position (vs usual care) | 1.00 (0.77 to 1.29) | 0.97 |
| Body mass index > 30 kg/m^2^ | 1.10 (0.82 to 1.47) | 0.54 |
| Initial SpO_2_ < 95% | 0.53 (0.40 to 0.71) | <0.0001 |
| Chronic hypertension | 0.69 (0.50 to 0.95) | 0.022 |
| Ischemic cardiomyopathy | 1.06 (0.61 to 1.87) | 0.83 |
| Type 2 diabetes | 0.77 (0.47 to 1.26) | 0.30 |
| Chronic obstructive pulmonary disease | 0.50 (0.23 to 1.10) | 0.08 |
| Immunosuppression | 0.65 (0.38 to 1.12) | 0.12 |

Abbreviation: sHR, subdistribution hazard ratio; SpO_2_, oxygen saturation measured by pulse oximetry

The time to clinical improvement (2 points on the WHO scale) was compared between groups by using a competing risk analysis approach (Fine and Gray model) with escalation of respiratory support (including increase in oxygen flow) as a competing event and adjusted for the stratification variables and baseline characteristics that were misbalanced between groups.

**PER-PROTOCOL ANALYSIS**

**eTable 3: Outcomes in per-protocol analysis**

|  | Prone position | Usual care | OR (95%CI) | P-value |
| --- | --- | --- | --- | --- |
|  | N= 42 | N= 131 |  |  |
| Primary outcome defined by non-invasive ventilation or tracheal intubation, or death within 28 days of enrollment, No. (%) | 3 (7.1) | 17 (13.0) | 0.71 (0.18 to 2.77) | 0.62 |
| Non-invasive ventilation, No. (%) | 2 (4.8%) | 8 (6.1) | 1.03 (0.18 to 5.90) | 0.98 |
| Intubation or death within 28 days, No. (%) | 2 (4.8%) | 14 (10.7%) | 0.65 (0.12 to 3.38) | 0.60 |
| Intubation within 28 days, No. (%) | 2 (4.8%) | 13 (9.9) | 0.65 (0.13 to 3.36) | 0.61 |
| Transfer to an intensive care unit within 28 days, No. (%) | 2 (4.8%) | 20 (15.3) | 0.32 (0.07 to 1.50) | 0.15 |

Abbreviations: aOR, odds ratios adjusted for stratification; CI, confidence interval.

**TIME-TO-EVENT EXPLORATORY ANALYSES**

Time-to-event analyses were used to further explore the potential differences between randomisation groups regarding the primary outcome (i.e., the occurrence of use of non-invasive ventilation, intubation or death with day 28 after inclusion) and the occurrence of use of non-invasive ventilation, the occurrence of intubation or death, and the occurrence of intubation separately.

For the primary outcome and the occurrence of intubation or death, the between-group relative risk (hazard ratio [HR]) was estimated in the framework of a Cox proportional-hazards model after checking the proportional-hazards assumption by scaled Schoenfeld residuals visual inspection. Because the patients were followed until day 28 after inclusion, the events of interest were collected for patients in this timeframe. Therefore, there were no competing events to consider. Between-group HRs were estimated.

The cumulative incidence of use of non-invasive ventilation was analysed by using a Fine and Gray model with the occurrence of intubation or death as competing risks. The cumulative incidence of intubation was analysed by using a Fine and Gray model with the occurrence of death as a competing risk. Between-group sub-distribution hazard ratios (sHRs) were estimated.

In each model, the interaction terms between the intervention and each of the stratification variables “initial SpO_2_” (< or ≥95% during a standardized test after randomisation) and the “patient’s body mass index” (< or ≥ 30 kg/m^2^) were introduced. To estimate HRs and sHRs, the stratification variables were systematically introduced in the model; the interaction terms were kept in the model only if statistically linked to the outcome at P<0.05.

Prespecified subgroup analyses according to the value of initial SpO_2_ and body mass index are provided regardless of the existence of statistically significant interactions.

Results are shown in the following figures.

**PRIMARY OUTCOME**

**eFigure 3: Whole study population:**

Note that the prone position group curve is slightly above the usual care group curve because the crude cumulative occurrence of treatment failure at 28 days was 14.1% in the prone position group and 12.9% in the usual care group. However, *hazard ratios and P values are those obtained through Cox proportional-hazards analysis adjusted for stratification (and here, for one interaction term).* This explains the apparent discrepancy between the aspect of the curves and the hazard ratio indicating that prone positioning may protect against treatment failure. Also note that the probability y axis has been truncated at 0.6 to make the curves distinguishable.

**eFigure 4**:

**A**: Subgroup with initial SpO_2_ < 95% **B**: Subgroup with initial SpO_2_ ≥ 95%

******

**PRIMARY OUTCOME**

**eFigure 5**:

**A**: Subgroup with body mass index <30 kg/m^2^ **B**: Subgroup with body mass index ≥30 kg/m^2^

**CUMULATIVE INCIDENCE OF NON-INVASIVE VENTILATION, with endotracheal intubation and death as competing risks**

**Figure S6:** Whole study population:

**eFigure 7***:*

**A**: Subgroup with initial SpO_2_ < 95% **B**: Subgroup with initial SpO_2_ ≥ 95%

**CUMULATIVE INCIDENCE OF NONINVASIVE VENTILATION, with endotracheal intubation and death as competing risks**

**eFigure 8***:*

**A**: Subgroup with body mass index <30 kg/m^2^  **B**: Subgroup with body mass index ≥30 kg/m^2^

**CUMULATIVE INCIDENCE OF INTUBATION, with death as a competing risk**

***e*Figure 9**: Whole study population

**eFigure 10***:*

**A**: Subgroup with initial SpO_2_ < 95% **B**: Subgroup with initial SpO_2_ ≥ 95%

**CUMULATIVE INCIDENCE OF INTUBATION, with death as a competing risk**

**eFigure 11***:*

**A**: Subgroup with body mass index <30 kg/m^2^ **B**: Subgroup with body mass index ≥30 kg/m^2^

**RISK OF INTUBATION OR DEATH**

**Figure S12**: Whole study population

***Results in the subgroup of patients with initial SpO_2_ < 95% and the subgroup of patients with initial SpO_2_ ≥ 95% are shown in the main manuscript***

**RISK OF INTUBATION OR DEATH**

**eFigure 13**:

**A**: Subgroup with body mass index <30 kg/m^2^ **B**: Subgroup with body mass index ≥30 kg/m^2^

**REFERENCES**

1. Nay MA, Planquette B, Perrin C, et al. Does awake prone positioning prevent the use of mechanical respiratory support or death in COVID-19 patients on standard oxygen therapy hospitalised in general wards? A multicentre randomized controlled trial: the PROVID-19 protocol. *BMJ Open*. 2022;12(7):e060320. doi:10.1136/bmjopen-2021-060320

2. Cao B, Wang Y, Wen D, et al. A Trial of Lopinavir–Ritonavir in Adults Hospitalised with Severe Covid-19. *N Engl J Med*. 2020;382(19):1787-1799. doi:10.1056/NEJMoa2001282
